# Co-registered Geochemistry and Metatranscriptomics Reveal Unexpected Distributions of Microbial Activity within a Hydrothermal Vent Field

**DOI:** 10.3389/fmicb.2017.01042

**Published:** 2017-06-13

**Authors:** Heather C. Olins, Daniel R. Rogers, Christina Preston, William Ussler, Douglas Pargett, Scott Jensen, Brent Roman, James M. Birch, Christopher A. Scholin, M. Fauzi Haroon, Peter R. Girguis

**Affiliations:** ^1^Department of Organismic and Evolutionary Biology, Harvard UniversityCambridge, MA, United States; ^2^Department of Chemistry, Stonehill CollegeEaston, MA, United States; ^3^Research and Development, Monterey Bay Aquarium Research InstituteMoss Landing, CA, United States

**Keywords:** hydrothermal vent, metatranscriptomics, diffuse flow, microbial activity, microbial ecology

## Abstract

Despite years of research into microbial activity at diffuse flow hydrothermal vents, the extent of microbial niche diversity in these settings is not known. To better understand the relationship between microbial activity and the associated physical and geochemical conditions, we obtained co-registered metatranscriptomic and geochemical data from a variety of different fluid regimes within the ASHES vent field on the Juan de Fuca Ridge. Microbial activity in the majority of the cool and warm fluids sampled was dominated by a population of *Gammaproteobacteria* (likely sulfur oxidizers) that appear to thrive in a variety of chemically distinct fluids. Only the warmest, most hydrothermally-influenced flows were dominated by active populations of canonically vent-endemic *Epsilonproteobacteria*. These data suggest that the *Gammaproteobacteria* collected during this study may be generalists, capable of thriving over a broader range of geochemical conditions than the *Epsilonproteobacteria*. Notably, the apparent metabolic activity of the *Gammaproteobacteria*—particularly carbon fixation—in the seawater found between discrete fluid flows (the intra-field water) suggests that this area within the Axial caldera is a highly productive, and previously overlooked, habitat. By extension, our findings suggest that analogous, diffuse flow fields may be similarly productive and thus constitute a very important and underappreciated aspect of deep-sea biogeochemical cycling that is occurring at the global scale.

## Introduction

Free-living microorganisms at hydrothermal vents play a fundamental role in supporting highly productive vent ecosystems and influence local, if not global, geochemistry (Resing et al., 2015). Since the discovery of hydrothermal vents in 1977 (Corliss et al., 1979), scientists have sought to better understand the ecology and physiology of hydrothermal vent microbes, particularly their phylogenetic and functional diversity, their rates of primary production, and their role in supporting the associated animal communities, either as free-living microbes upon which animals graze or as symbionts. To date, most of the studies on free-living microbes have focused on communities associated with the prominent high temperature chimneys (200–400°C) and their fluid plumes, which can be tens of meters and hundreds of meters tall, respectively (e.g., Klinkhammer and Hudson, 1986; Cowen et al., 1990; Kadko et al., 1995). Many studies have also focused on the less conspicuous lower temperature (<100°C) diffuse vents, which account for at least half of the total thermal and chemical flux into the overlying ocean (Rona and Trivett, 1992; Baker et al., 1993; Bemis et al., 1993; Elderfield and Schultz, 1996; Wankel et al., 2011). Previous studies have shown that diffuse flows foster the growth of microbial communities derived from both the overlying seawater (Anantharaman et al., 2013; Anderson et al., 2013; Dick et al., 2013, and references within) and the subsurface (e.g., Akerman et al., 2013; Fortunato and Huber, 2016). The lower temperatures and mixing of oxygen-rich seawater with chemically-reduced hydrothermal fluids should yield a diversity of niches for microbes, and thus support higher rates of primary productivity than high temperature vents (Perner et al., 2011; Olins et al., 2013).

Despite a wealth of research on diffuse flow vents (e.g., Bemis et al., 2012; Akerman et al., 2013; Campbell et al., 2013), several key questions remain. Specifically, little is known about the fate of the fluids that emanate from diffuse vents—and accordingly, the phylogenetic and functional characteristics of the associated microbial communities as they disperse into the environment. While it has been suggested that diffuse flows lead to plumes that stay lower in the water column, pushed laterally along the vent field by bottom currents (Rona and Trivett, 1992; Pruis, 2004; Hautala et al., 2012), to our knowledge no geochemical studies have characterized the presence and structure of such plumes, nor have studies determined whether vent-derived endemic microbes are dispersed and remain active within these plumes. Previous studies of the microbial communities associated with warm diffuse (<100°C) flows, in particular at the Axial Volcano vent fields, have demonstrated that diffuse flows are commonly occupied by abundant, active populations of *Epsilonproteobacteria* and *Gammaproteobacteria*, all of which may be engaged in chemoautotrophy (Huber et al., 2002, 2003, 2006; Ver Eecke et al., 2012; Akerman et al., 2013; Anderson et al., 2013; Meyer et al., 2013; Fortunato and Huber, 2016). The environmental factors that govern the abundance and activity of these and other microbial taxa, however, have not been determined.

This work attempts to address two questions: First, to what extent are hydrothermally-derived compounds found in the waters surrounding diffuse flows? And second, what is the activity and distribution of vent-endemic and non-endemic microbes throughout these waters, as well as within the diffuse flow fluids? To this end, we collected samples for geochemical analyses and metatranscriptomic library construction from an extensive survey of fluids within and around diffuse hydrothermal flows at the ASHES vent field (Butterfield et al., 2004) in the Axial volcano. As part of that effort, we deployed the Deep-sea Environmental Sample Processor, or D-ESP, a state-of-the-art, autonomous microbial analytical laboratory (Ottesen et al., 2011; Pargett et al., 2013; Ussler et al., 2013), to collect, filter, and preserve six RNA samples *in situ* for metatranscriptomics from one diffuse flow and one intra-field site. We also collected ten additional fluid and particulate samples from five adjacent sites spanning three habitat types: diffuse hydrothermal flows (in this work all <40°C) where there was visible shimmering and microbial mats (hereafter, “diffuse”), intra-field fluids found among the diffuse vents (~2.5°C), but without any visual biotic or abiotic indications of fluid flow (hereafter, “intra-field”), and background seawater collected hundreds of kilometers outside of Axial Caldera (hereafter, “background seawater”) using a Niskin water sampler. In all cases, we made co-registered geochemical measurements via *in situ* mass spectrometry or shipboard geochemical assays to provide environmental context.

With these samples, we considered the resulting metatranscriptomic and geochemical data from three perspectives: (1) a dataset-wide investigation of the most abundant transcripts that provide a broad view of the active community and their gene expression profiles in different habitat types; (2) a targeted analysis of gene expression relevant to chemosynthesis and elemental cycling; and (3) differential expression analysis of samples collected using the D-ESP to identify the most differentially-expressed genes between our most hydrothermally-influenced diffuse flow site and the intra-field fluids. The data presented here confirm previous findings (e.g., Akerman et al., 2013) that microbial activity at many ASHES vent field diffuse flows is dominated by *Gammaproteobacteria* sulfur oxidizers (GSOs). However, our findings also demonstrate that low temperature diffuse flow vents in a single vent field can have dramatically different profiles of microbial activity dominated by vent-associated *Epsilonproteobacteria*. Importantly, these data reveal that the intra-field habitat represents a zone of microbial activity—and likely significant source of primary productivity—that is geochemically distinct from that of either seawater or hydrothermal flows. This intra-field habitat is analogous to plumes from high temperature chimneys, in which chemoautotrophic *Gammaproteobacteria* are abundant, active, and potentially contribute substantially to net hydrothermal vent productivity.

## Materials and methods

### Study site

**Figure 1:**
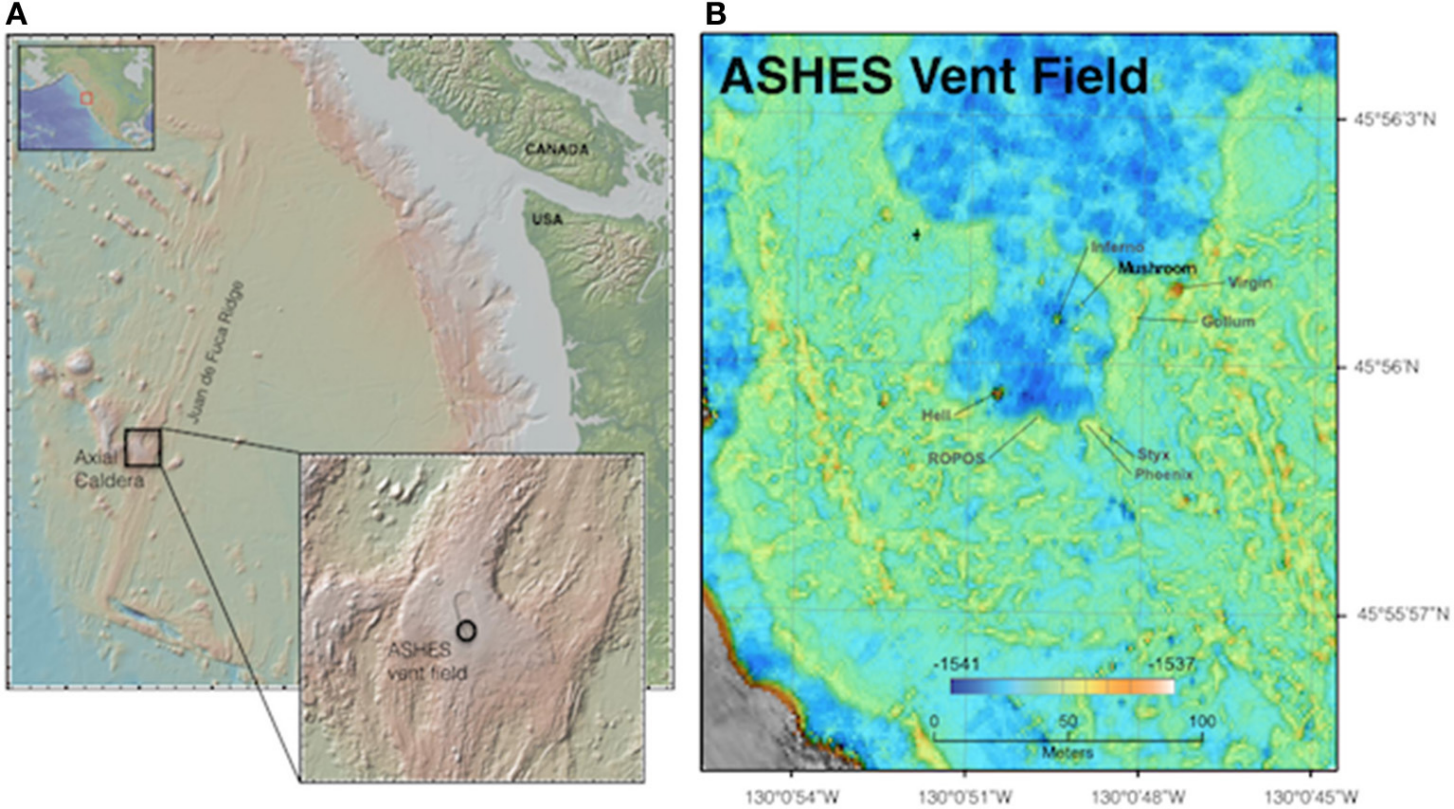
Maps of the Juan de Fuca Ridge (A) showing location of Axial Caldera and ASHES vent field, and (B) ASHES vent field showing the location of Mushroom chimney, identifying our study sites.

The Axial Seamount Hydrothermal Emissions Study (ASHES) vent field (45° 56′ N, 130° 31′ W; Figure 1) contains a diversity of hydrothermal fluid flow types including an abundance of diffuse venting (Butterfield et al., 2004). An area roughly 200 m by 1,200 m, ASHES is located adjacent to the western wall of the Axial Volcano caldera along the Juan de Fuca Ridge. Patchy, diffuse venting is common throughout ASHES along the cracks in the seafloor, and discrete venting occurs at isolated sulfide chimneys as well as anhydrite mounds (Rona and Trivett, 1992).

### Sample collection

**Figure 2:**
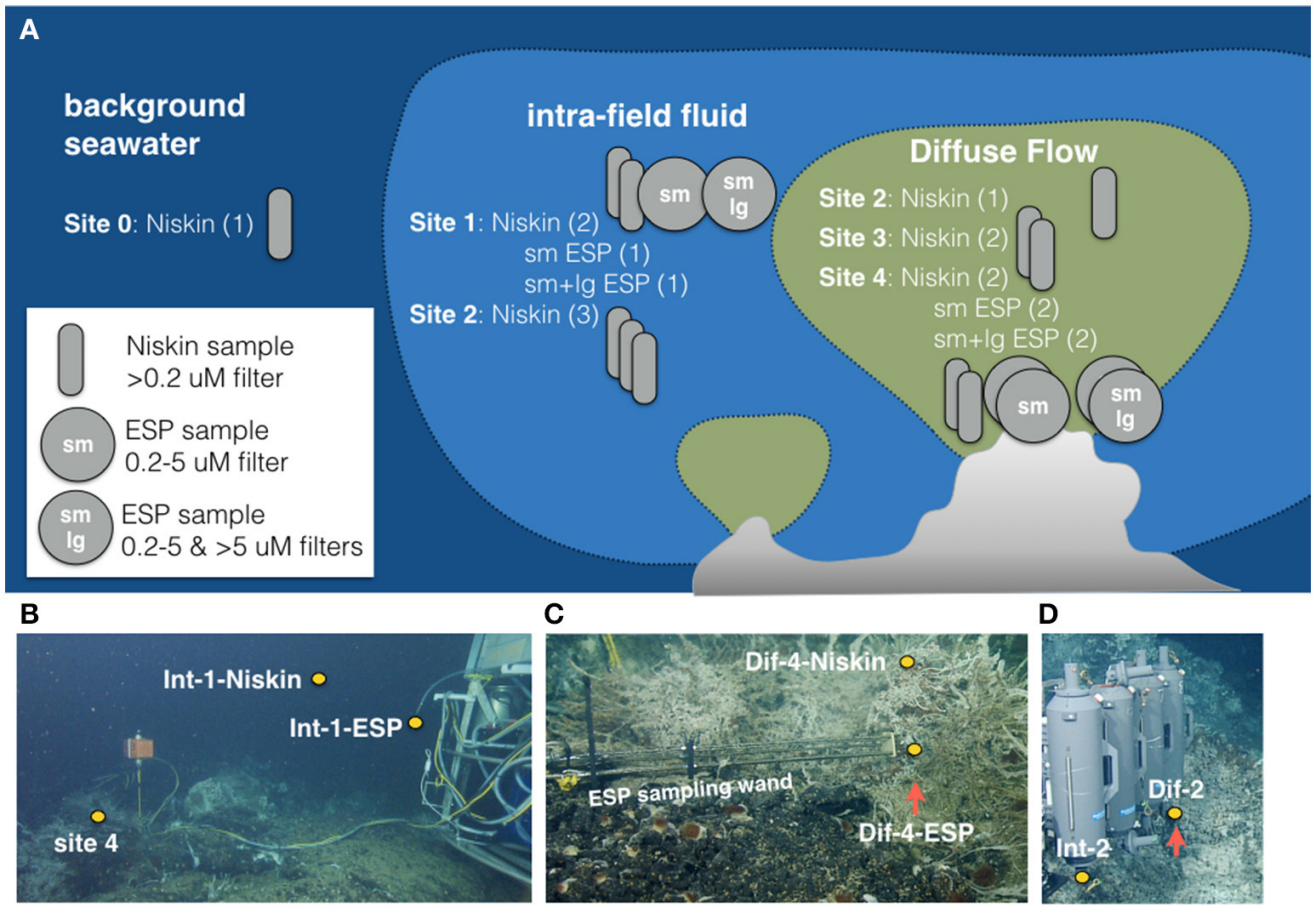
**(A)** Schematic of samples collected for this work at 5 sites (0–4) representing three habitat types (seawater, intra-field, and diffuse flow) using two sampling methods (Niskin and Deep Environmental Sample Processor ESP). **(B)** ESP deployed at sites 1 and 4 showing position of samples collected at Site 1 with Niskin and ESP. **(C)** ESP sampling wand deployed at Site 4 showing positions of samples collected with Niskin and ESP. **(D)** Site 2 Niskin samples being collected showing position of intra-field and diffuse samples collected at Site 2.

Sampling occurred from July 14–21, 2011, aboard the R/V *Western Flyer* using the ROV *Doc Ricketts* (Monterey Bay Aquarium Research Institute). Samples were collected using the Deep-sea Environmental Sample Processor (D-ESP), as well as with ROV-mounted Niskin bottles (overviewed in Figure 2A, detailed description below). The D-ESP is a self-contained robotic laboratory that collects and recovers particles from fluid samples *in situ* for molecular microbiological analyses (Ottesen et al., 2011; Pargett et al., 2013; Ussler et al., 2013). The D-ESP was deployed near a diffuse flow vent at the base of Mushroom chimney, (~45° 56.0011′ N, 130° 0.8218′ W; Figure 1), and was programmed to collect replicate samples over a 4-day deployment (see Supplemental Table 1 for sampling schedule). Samples were collected as in Ussler et al. (2013) with the addition of an extendable sampling wand that enabled sampling directly from a diffuse vent (Figure 2C). A second sample inlet, located on the D-ESP 1 m off the bottom and ~3 m away from the diffuse vent, was used to sample the intra-field (within vent field between diffuse flows) water (Figure 2B; Pargett et al., 2013). The D-ESP pumped 5 L of water from one inlet at a time into a decompression module. One liter of sample was filtered through stacked 5 and 0.2 μm Durapore filters (referred to throughout as sm and lg respectively), and immediately preserved in RNALater™ (Ambion Inc.). Preserved samples were stored within the D-ESP at ~8°C for 5–14 days before recovery and subsequently stored at −80°C until processing. Additional details about D-ESP operations can be found in Supplemental Materials Methods.

Fluids were also collected using 5 L Niskin bottles affixed to a swing-arm on the ROV (Figure 2D). The Niskin bottles were positioned by the ROV over the diffuse flows, and samples were collected only when shimmering water was clearly seen exiting the top of the bottle. Niskin bottles were also used to collect fluids from near both D-ESP inlets, which enabled direct comparison of D-ESP and Niskin-collected samples. Upon ship-board retrieval, water from Niskins was filtered onto 0.2 μm Durapore filters (1 L per filter), immediately preserved in RNALater™, and stored at −80°C until processed. The time elapsed between Niskin sample collection and ROV retrieval on ship was between 3 and 9 h, and process time for discrete samples collected by Niskin bottles was 1–2 h.

In this work “Site” refers to one of the five sampling locales, named Sites 0–4. Site 0 represents background seawater, collected from 3 km outside the Axial volcano caldera and ~2 m above the seafloor. Sites 1–4 were within ASHES vent field and represented diffuse flows (Sites 3 and 4), intra-field waters (Site 1), or a location where both environments were sampled (Site 2). Sites 1 and 4 were located near the base of Mushroom chimney (Figure 1) and Sites 2 and 3 were each located ~10 m away from Sites 1 and 4.

A sample refers to a discrete water sample that was collected and filtered either onto a single 0.2 μm filter (Niskin) or stacked 5 μm (lg) and 0.2 μm (sm) filters (D-ESP). Between one and four samples were collected at each site. Individual metatranscriptomes described herein come from a single filter and therefore represent a single >0.2 μm Niskin sample or either a 0.2 or 5 μm size fraction D-ESP sample. Eleven Niskin and nine D-ESP filters were analyzed for this study. Because the D-ESP samples were passed through two stacked filters, six of the D-ESP filters were paired 0.2 and 5 μm filters (representing three discrete collections), while the additional three D-ESP filters were 0.2–5 μm size fractions (the corresponding 5 μm filter fraction was not sequenced). The Niskin filters, in contrast, represent an entire sample filtered (>0.2 μm). See Table 1 for more detail. In total, paired microbiological and chemical analyses were completed at five sites (Sites 0–4; see Table 1).

**Table 1:** Description of samples and filters sequenced.

**Sample ID**	**Sampling method**	**Site No**.	**Environment classification**	**Number of filters sequenced**
SW-Niskin	Niskin	0	Non-vent field background (seawater)	1
Int-1-Niskin	Niskin	1	Vent field background (intra-field)	2
Int-1-lg-ESP Int-1-sm-ESP	D-ESP	1	Vent field background (intra-field)	1 >5 um (lg) 2 0.2–5 um (sm)
Int-2-Niskin	Niskin	2	Vent field background (intra-field)	3
Dif-2-Niskin	Niskin	2	Diffuse flow	1
Dif-3-Niskin	Niskin	3	Diffuse flow	2
Dif-4-Niskin	Niskin	4	Diffuse flow	2
Int-4-lg-ESP	D-ESP	4	Diffuse flow	2 > 5 um (lg)
Int-4-sm-ESP				4 0.2–5 um (sm)

### Geochemical analyses

A Seabird 16plus conductivity, temperature, depth (CTD) sensor and an Aanderaa oxygen optode (model 3830) placed in line with the D-ESP measured oxygen, salinity, and temperature throughout the entire deployment. Methane concentrations were determined by collecting 250 mL subsamples from Niskin bottles in gastight serum vials and analyzing these shipboard with a gas chromatograph (as in Ussler et al., 2013). Ten milliliter subsamples from the Niskins were also filtered (through 0.2 μm Durapore filters) for major and minor element analyses, acidified in hydrochloric acid, and frozen at −80°C. Major and minor elements (including Mg, Mn, Fe, and Si) were quantified using Inductively Coupled Plasma-Atomic Emission Spectrometer (ICP-AES; as in Wheat et al., 2010). Ten milliliter Niskin subsamples were also preserved for H_2_S analyses in 1 M zinc acetate (Zn(C_2_H_3_O_2_)_2_) stored at 4°C, and quantified via a colorimetric assay on a Spectramax Plus 384 absorbance microplate reader (Molecular Devices, LLC; Cline, 1969).

### Nucleic acid extraction

For both D-ESP and Niskin samples, filters were rinsed with TE buffer to remove RNALater™ (Ambion, Inc.), and the cells were lysed by incubating filters with Trizol™ (Life Technologies, Inc.) for 20 min at 25°C then 10 min at 85°C. Nucleic acids were separated using 24:1 chloroform:isoamyl alcohol. RNA was precipitated in 100% isopropanol with 100 mg of Glycoblue™ (Ambion, Inc.) to improve recovery. RNA was resuspended in RNA Storage buffer (Ambion, Inc.). Residual DNA was removed with TURBO DNase™ (Ambion, Inc.) per the manufacturer's instructions. RNA was quantified with a Qubit 2.0 fluorometer (Life Technologies, Inc.) using the RNA high specificity assay (Life Technologies, Inc.).

### Metatranscriptomic sequencing

RNA sample quality control, Illumina TruSeq Stranded mRNA sample preparation (without rRNA removal), and Illumina HiSeq2000 101 cycle paired-end sequencing were carried out by the University of Utah Microarray Core Facility (Salt Lake City, UT; see Supplemental Materials Methods for details). To constrain the extent of bias between the two lanes used for sequencing, a subset of samples from the first sequencing run were re-run on the second lane. Statistical analyses of taxonomic and functional groups did not show any significant differences between sequencing runs (data not shown).

### Global metatranscriptomic analyses

Sequence data from all 20 metatranscriptomic libraries were analyzed in MG-RAST (http://metagenomics.anl.gov/) using default quality control parameters at submission, with the exception of running Duplicate Read Inferred Sequencing Error Estimation (DRISEE), which is designed to remove artificial duplicate read sequences, but can inadvertently eliminate biologically-relevant sequences from Illumina data (Eren et al., 2014). Metatranscriptomes had between 9.4 and 20.8 million sequences after MG-RAST quality control (values in Supplemental Table 2). For analysis of sequences within MG-RAST, the following parameters were selected: maximum *e*-value cutoff of 10, minimum percent identity of 80%, and minimum alignment length cutoff of 25. The non-redundant multisource ribosomal RNA annotation (M5RNA) database was used for taxonomic classifications using Best Hit classification (which includes all identical 100% annotations for each read), and SEED and Subsystem classification were used for functional gene identification. Read abundances for genes of interest were manually curated by searching for variations of gene names and then compiled. Hit numbers were normalized to total functional or domain-level taxonomic hits. Metatranscriptomes ranged from 7.1 to 22.3 million taxonomic hits, 1–2.5 million SEED hits, and 44–645 thousand L1 Subsystem hits (see Supplemental Table 2 for values). Metatranscriptomes are available on MG-RAST (http://metagenomics.anl.gov/mgmain.html?mgpage=project&project=mgp4026, Project ID mgp4026).

### Targeted *De novo* metatranscriptomic assembly and differential expression analyses

A subset of six filters was selected for targeted differential expression analyses between one diffuse and one intra-field site. The selected filters consisted of all D-ESP samples where both the 0.2 and 5 μm filters were extracted and sequenced (one intra-field Site 1 sample and two diffuse Site 4 samples). These samples were selected to minimize variables introduced by different sampling methods (see **Figure 9** for functional categories that differed by sampling method and **Figure 4B** for diversity comparison by sampling method) and to target the ends of the geochemical spectrum of sites sampled. In total, six metatranscriptomic libraries representing three fluid samples, one from Site 1 (intra-field) and two from Site 4 (diffuse flow) were used in this analysis.

Metatranscriptomic reads were trimmed and adapters were removed using Trimmomatic (Bolger et al., 2014) with the following parameters: -LEADING:3, -TRAILING:3, -SLIDINGWINDOW:4:15, -MINLEN:50. RiboPickr (Schmieder and Edwards, 2011) was used to remove ribosomal RNA reads prior to assembly of functional transcripts (80% alignment coverage threshold, 90% alignment identity threshold, using the non-redundant ribosomal RNA database; 4/15/2015 update version).

Metatranscriptomic functional paired reads from all six libraries were assembled *de novo* using Trinity (Grabherr et al., 2011); minimum contig length of 50. Differential expression between Site 1 and Site 4 was calculated using DESeq2 (Love et al., 2014) in R using the trans.counts.matrix file generated in Trinity. Results were filtered by adjusted *p* > 0.05, and for contigs that had 100 or more reads from the Site 1 sample (consisting of the two paired filters) or 200 or more reads from the two Site 4 samples (from four paired filters). The remaining differentially-expressed contigs were identified using blastx (Altschul et al., 1990) on NCBI's nr database (Pruitt, 2005; version updated April 15, 2015) using default parameters.

Transcripts from the resulting list were manually pooled according to replicate functions (i.e., all ribosomal proteins). Log odds ratios were calculated to compare relative over- and under-expression of this curated transcript list between the two sites.

### Statistical analyses

To statistically examine the patterns in microbial gene expression, we posed five testable scenarios that could explain patterns in gene expression. Differences could be not significant (null hypothesis) or they could be significant as a function of (1) site, (2) environment, (3) an outlier single site (one that is distinct from all the others), (4) sampling method. To test which of these scenarios best explained the variation in our data, we grouped samples accordingly (i.e., by Sites—1–4, by environment—diffuse flow or intra-field fluid, by separating out the most hydrothermally influenced Site—Sites 1–3 vs. Site 4, or by method—Niskin vs. ESP) and performed statistical tests in R (Mann Whitney U or Kruskal-Wallis for four or two groups respectively). We used *p* = 0.05 for a significance cut-off after correcting for multiple comparisons. Gene categories that were significant after correction with the conservative Bonferroni correction were considered highly significant, while those significant after the less conservative Benjamini-Hochberg test were considered significant. Site 0 (seawater) was not used in statistical analyses because it was represented by a single sample (see Table 1 for number of samples in each grouping). These statistical tests were performed on the selected genes of interest as well as the SEED subsystem categories. See Supplemental Table 3 for *p*-values.

Cluster analysis (**Figure 5**) was carried out in R on taxomonic IDs with singleton and doubletons removed. The vegdist function in the vegan package was used to calculate Bray-Curtis dissimilarity matrix, and clustering was carried out using the hclust function and the complete linkage method.

## Results

### Fluid geochemistry

**Figure 3:**
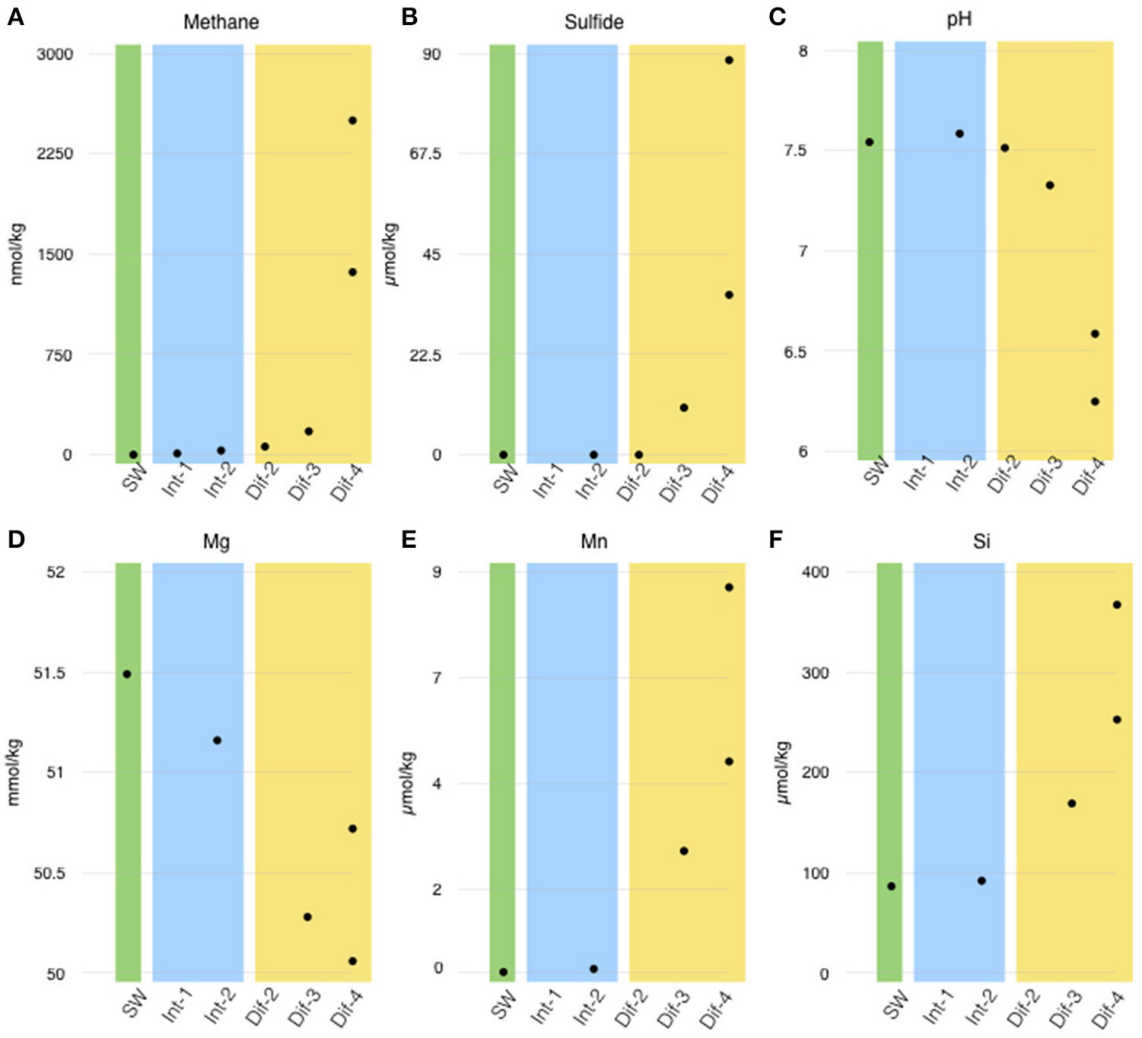
Chemical measurements corresponding to sequenced filters (site numbers noted in X-axis). **(A)** Methane, **(B)** sulfide, **(C)** pH, **(D)** magnesium, **(E)** manganese, and **(F)** silica concentrations are arranged by habitat type; green—seawater (SW), blue—intra-field (Int), and yellow—diffuse flow (Dif). Note than Mn and Si were not measured at Site 2.

The diffuse flow, intra-field, and background fluids recovered for analyses showed a wide range of geochemical composition (Table 2). Diffuse flow samples (from Sites 2, 3, and 4) showed the highest variation in temperature and concentrations of vent-derived compounds such as methane (which varied from 19.25 to 2558.96 nmol/l) and sulfide (which varied from non-detectable to 90.61 μM). The only fluids with pH below 7 came from diffuse flows. However, other chemical markers of hydrothermal flow, such as increased silica and manganese and decreased magnesium relative to seawater, show marked hydrothermal influence in just some of the diffuse flows. The background (Site 0) sample, unsurprisingly, showed no evidence of any hydrothermal influence in chemical composition, temperature, or pH. Notably, the intra-field fluids were in some ways indistinguishable from the background sample. The intra-field and background fluids showed no significant difference in silica, sulfide, manganese concentrations, or pH (Figure 3, Table 2). However, methane (and in some cases, magnesium) showed a marked difference in concentration between the intra-field fluids and the background seawater (methane averaged 21.8 nmol/kg in the intra-field water, well above the 0.55 nmol/kg typically found in seawater). Geochemical measurements associated with the samples that were sequenced show that Site 2 intra-field (Int-2) and diffuse (Dif-2) samples shared similar chemistry and that diffuse samples from Sites 3 and 4 had increasing hydrothermal influence (Figure 3). Additional geochemical measurements from samples that were not sequenced are shown in Supplemental Figure 1. Among the diffuse flow samples, methane, sulfide, silica, and manganese increase from Site 2 to Site 3 to Site 4, indicating increasing hydrothermal influence in these samples. pH correspondingly decreased among these samples.

**Table 2:** Geochemical data (including samples not sequenced).

			**Mg**	**Mn**	**Si**	**pH**	**Sulfide**	**CH4**
**Dive**	**Niskin**	**Sample type**	**mmol/kg**	**micromol/kg**	**micromol/kg**		**micromolar**	**nmoles/liter**
DR-261	Niskin 7	seawater	51.38	0.04	97.60	7.54	0.00	0.53
DR-261	Niskin 5	seawater	51.60	0.03	67.90	nm	0.00	0.57
DR-261	Niskin 6	seawater	51.49	0.00	86.60	nm	0.00	0.57
DR-261	Niskin 8	seawater	nm	nm	nm	nm	nm	0.53
		Seawater average	51.49	0.02	84.03	7.38	0.00	0.55
		Seawater St. Err.	0.06	0.01	8.67		0.00	0.01
		Seawater n	3	3	3	1	3	4
ESP upcast	ESP upcast	Intra-field	50.61	0.98	120.70	7.21	0.00	nm
ESP upcast	ESP upcast	Intra-field	50.94	0.93	119.60	nm	0.00	nm
DR-258	Niskin 5	Intra-field	50.72	0.19	97.60	7.63	0.00	29.84
DR-259	Niskin 5	Intra-field	nm	nm	nm	nm	nm	4.79
DR-259	Niskin 6	Intra-field	nm	nm	nm	nm	nm	7.48
DR-259	Niskin 7	Intra-field	nm	nm	nm	7.65	0.00	26.82
DR-259	Niskin 8	Intra-field	nm	nm	nm	7.51	0.00	25.13
DR-254	Niskin 8	Intra-field	nm	nm	nm	nm	nm	13.31
DR-252	Niskin 5	Intra-field	nm	nm	nm	nm	nm	10.12
DR-254	Niskin 5	Intra-field	nm	nm	nm	nm	nm	46.20
DR-257	Niskin 5	Intra-field	51.16	0.07	92.10	7.58	0.00	32.47
		Intra-field ave	50.86	0.54	107.50	7.52	0.00	21.80
		Intra-field St. Err.	0.16	0.04	1.94	0.03	0.00	4.58
		Intra-field n	4	4	4	5	6	9
DR-257	Niskin 7	diffuse	51.27	0.15	85.50	7.52	0.00	19.25
DR-258	Niskin 6	diffuse	50.06	4.74	252.70	6.59	36.73	1398.30
DR-258	Niskin 7	diffuse	50.72	8.65	367.10	6.25	90.61	2558.96
DR-258	Niskin 8	diffuse	50.28	2.72	169.10	7.33	10.81	179.51
DR-254	Niskin 7	diffuse	nm	nm	nm	nm	nm	120.91
DR-252	Niskin 6	diffuse	nm	nm	nm	nm	nm	638.99
DR-252	Niskin 7	diffuse	nm	nm	nm	nm	nm	657.19
DR-252	Niskin 8	diffuse	nm	nm	nm	nm	nm	383.84
DR-257	Niskin 8	diffuse	nm	nm	nm	nm	nm	62.57
		Diffuse average	50.58	4.06	218.60	6.92	34.54	668.84
		Diffuse St. Err.	0.27	1.79	60.13	0.30	20.22	276.79
		Diffuse n	4	4	4	4	4	9

*Mg, Mn, and Si represent total elemental concentration. Nm, not measured. Shaded rows represent summary data for each habitat type*.

Finally, via the *In Situ* Ultraviolet Spectrophotomoter (ISUS) run concurrently with the D-ESP sampling, oxygen was found to be substantially lower in the Site 4 diffuse flow (0.03 ml L^−1^) than in the intra-field water at Site 1 (0.96 ml L^−1^). The temperature was substantially higher at the Site 4 diffuse flow (34.2°C) than the intra-field water at Site 1 (2.5°C; see Supplemental Figure 5A). While oxygen and temperature measurements were only available for the D-ESP samples, if we assume linear mixing between seawater and vent fluid (as in Baker et al., 1993), we can conclude that temperature and oxygen concentration of the discrete samples (i.e., at Sites 2 and 3) would fall between those measured at Sites 1 and 4.

### Composition of the active microbial community

**Figure 4:**
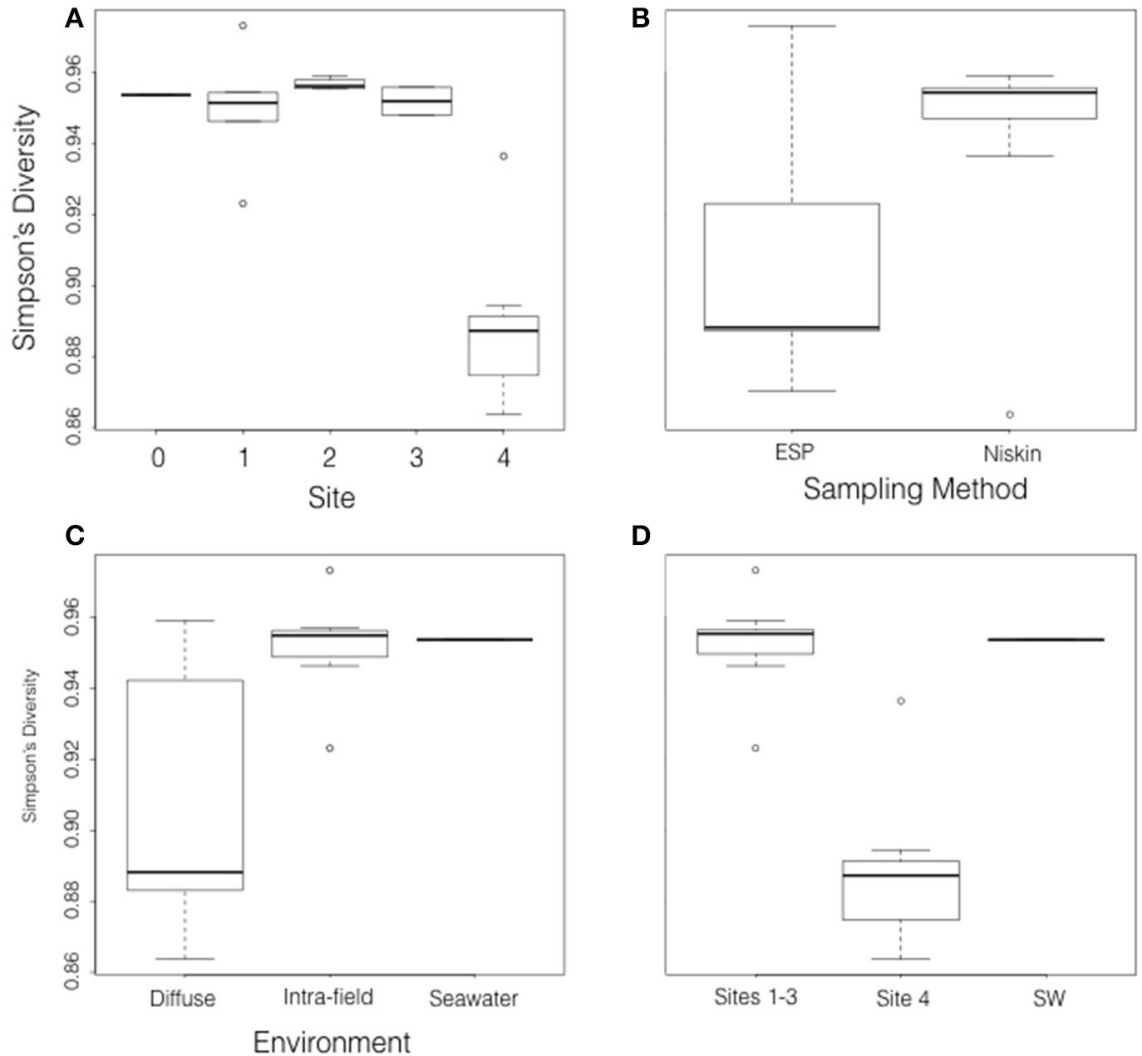
Box and whisker plots showing Simpson's Diversity of the 20 metatranscriptomes grouped by **(A)** site, **(B)** sampling method, **(C)** habitat type, and **(D)** cluster analysis (Figure 5) grouping.

**Figure 5:**
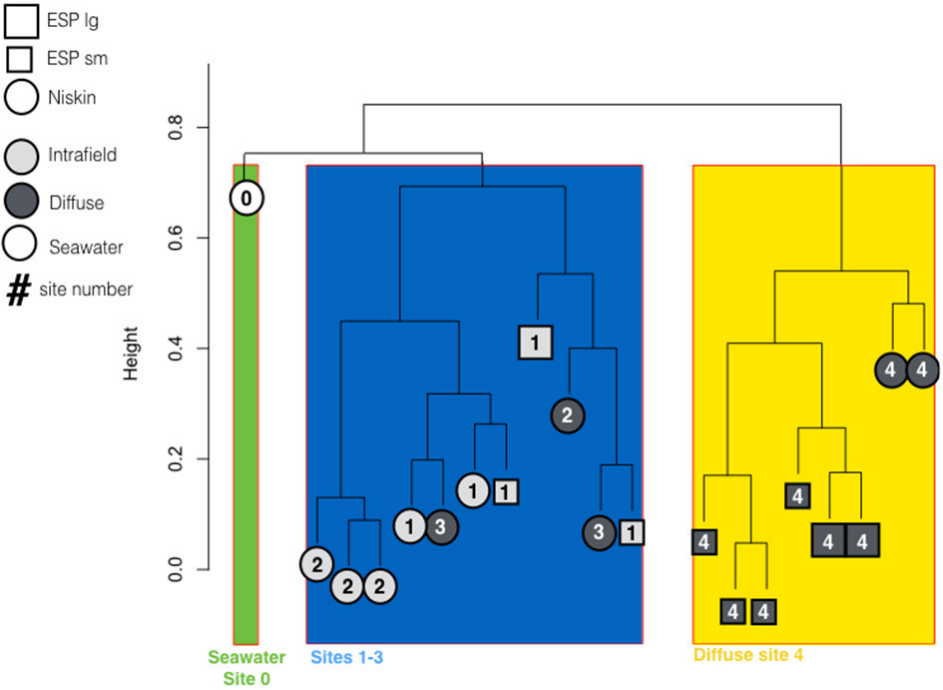
Cluster diagram generated with Complete Linkage method showing similarity of metatranscriptomes based on identified rRNA reads not including singleton or doubleton reads. Major groupings show seawater, Site 4 diffuse flows, and Sites 1–3 samples as distinct.

**Figure 6:**
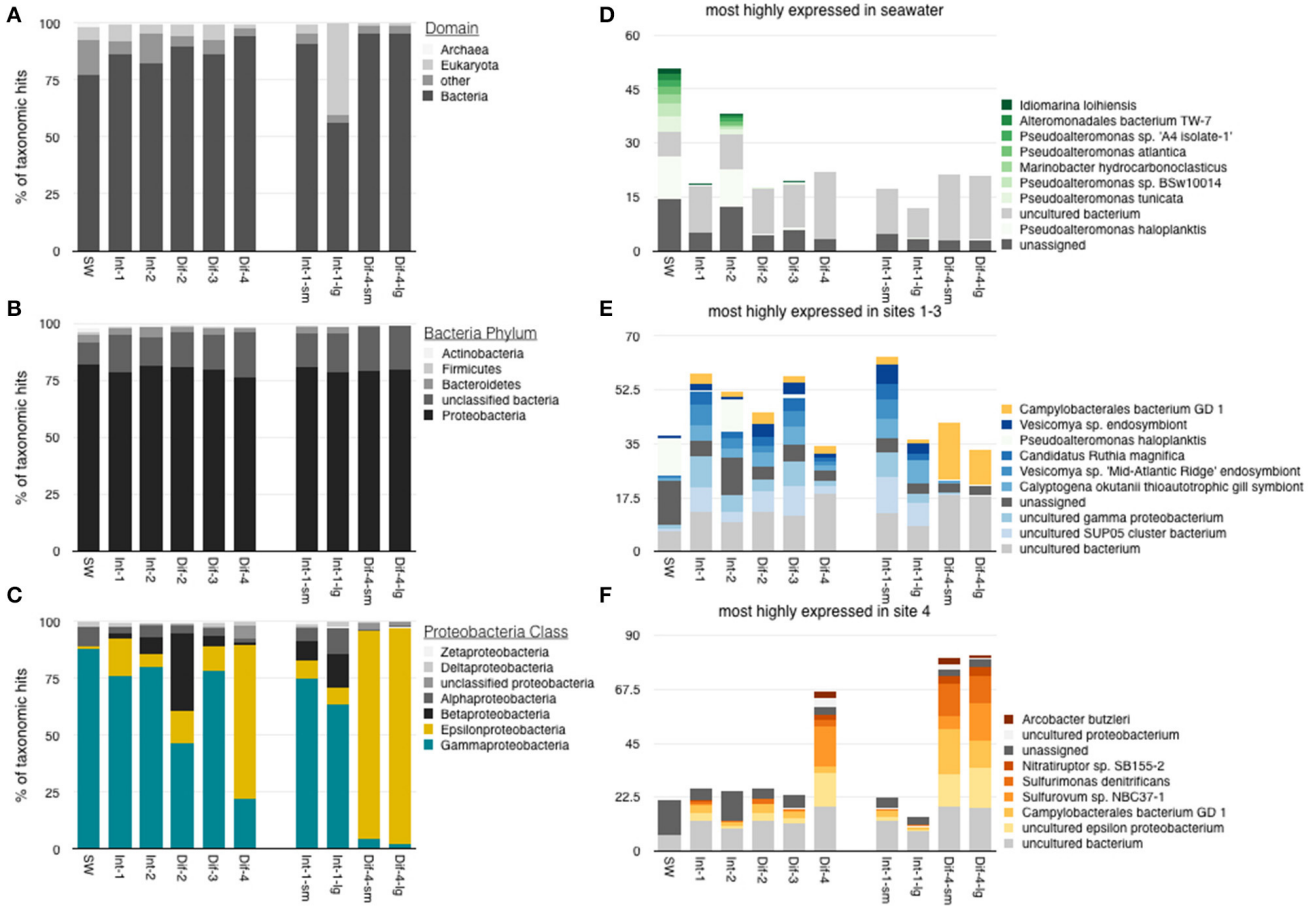
Taxonomic identification of rRNA reads, averaged by sample type. **(A)** Domain-level distribution of all rRNA identifications, **(B)** phylum-level distribution of bacterial reads, and **(C)** class-level identification of proteobacterial reads. 10 most abundant genus-level identifications from **(D)** seawater, **(E)** Sites 1–3, and **(F)** Site 4 (note hits that appear in two of d, e, and c are among most abundant in both of those categories).

Measurements of Simpson's Diversity indicate that active communities from Site 4 were less diverse than those from sites 1–3, and that differences in diversity were more obvious when the 20 metatranscriptomes were grouped by site number (Figure 4A), than when grouped by sampling method (Figure 4B), or habitat type (Figure 4C). Other diversity metrics showed similar results (data not shown). Cluster analysis grouped the samples into three groups based on similarity: the seawater sample, all samples from Site 4, and the remaining samples from Sites 1–3 (Figure 5; NMDS plot of same data found in Supplemental Figure 2). The identified rRNA from all samples was dominated by bacterial sequences (Figure 6A), which represented 56–96% of each sample's identified rRNA reads. Individual replicate filters were similar, and so we present the averages of all replicates of the same filter type (e.g., all 0.2–5 μm D-ESP filters from Site 4) in these figures. Overall, 99.29% of taxonomic hits were >97% identical, and 95.50% of hits were >99% identical.

Bacterial communities were all dominated by Proteobacteria (Figure 6B). *Gammaproteobacteria* in particular dominated the rRNA transcripts from all samples except those collected from Site 4 (Figure 6C). The most abundant *Gammaproteobacteria* rRNA reads in the seawater sample were from the genera *Idomarina, Alteromonadales, Marinobacter*, and *Pseudoalteromonas* (Figure 6D). In contrast, the most highly represented *Gammaproteobacteria* from Sites 1–3 were closely aligned to the SUP05 clone or the symbionts of vent animals (Figure 6E).

The metatranscriptomic libraries from the most hydrothermally-influenced diffuse flow (Site 4) revealed an active, autotrophic community dominated by vent-associated *Epsilonproteobacteria* such as *Sulfurovum* and *Sulfurimonas* (Figure 6C). The most highly represented taxa within this class were from the genera *Arcobacter, Nitratiruptor, Sulfurimonas, Sulfurovum*, and *Campylobacteriales* (Figure 6F). These taxa are known to be vent associated and typically found in warmer, less oxic vent habitats (Huber et al., 2010; Yamamoto and Takai, 2011; Wright et al., 2012; Akerman et al., 2013; Anderson et al., 2013; Meyer et al., 2013).

### Broad functional characterization

**Figure 7:**
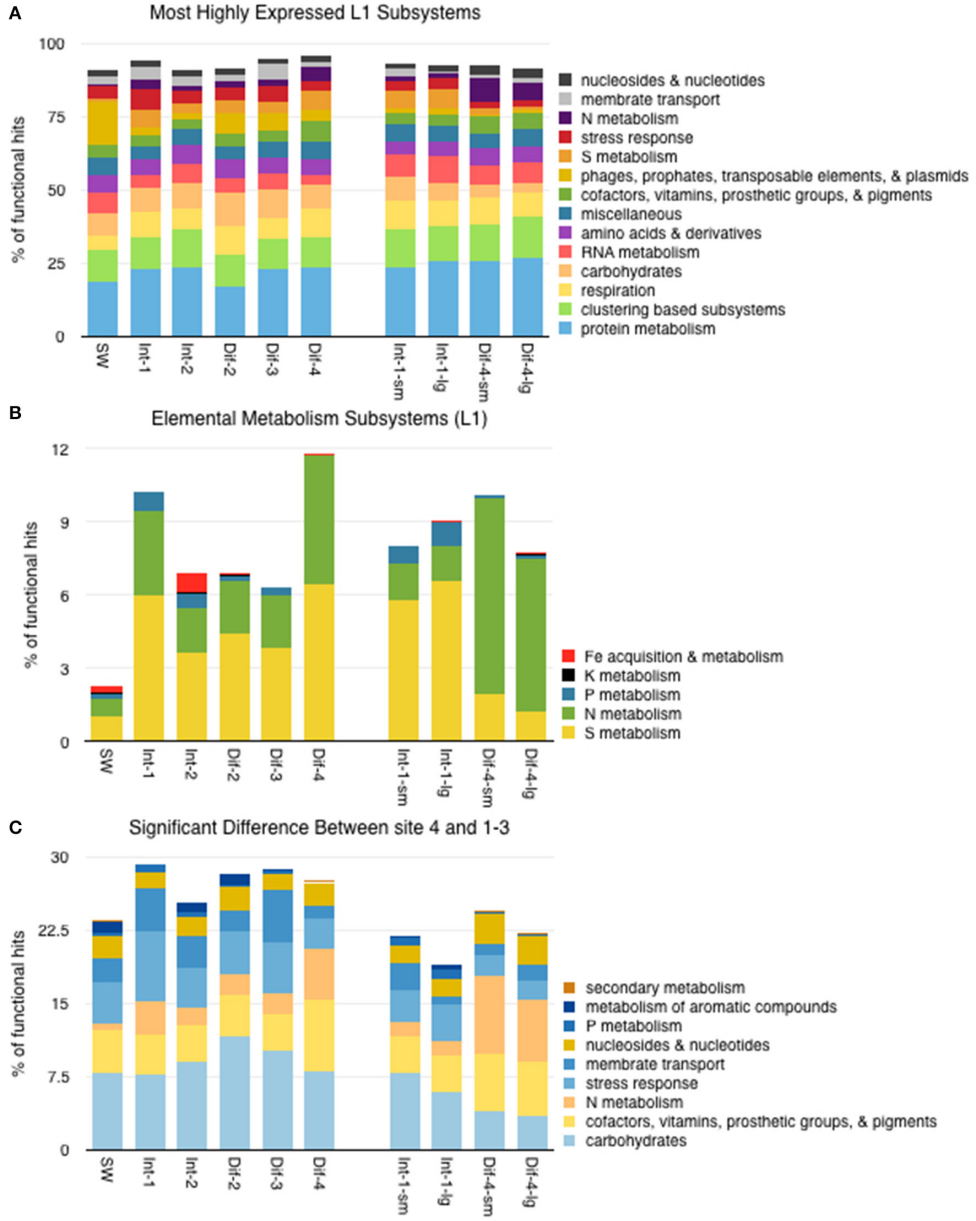
**(A)** Distribution of functional reads assigned to the most highly expressed Level 1 SEED subsystems. **(B)** Distribution of functional reads assigned to Level 1 subsystems specific to elemental metabolism. **(C)** Distribution of functional reads of Level 1 subsystems that showed significantly different expression between Sites 1–3 and Site 4. See Supplemental Table 3 for *P*-Values.

Hierarchical SEED Subsystem functional gene classification showed that all samples had a fairly similar, broadly categorized (Subsystem Level 1, L1) profile of transcriptional activity. Most highly expressed categories are shown in Figure 7A. There were notable differences, however, in categories of elemental metabolism (Figure 7B). Some L1 subsystem categories did show statistically significant differences in expression between Sites 1–3 and Site 4 (Figure 7C). The following L1 subsystems were more highly expressed in Sites 1–3 than Site 4: metabolism of aromatic compounds; P (phosphorus) metabolism; membrane transport; stress response; and carbohydrates. In contrast, the following L1 subsystems were more highly expressed in Site 4: secondary metabolism; nucleosides and nucleotides; N (nitrogen) metabolism; and cofactors, vitamins, prosthetic groups & pigments.

### Expression of selected functional genes

**Figure 8:**
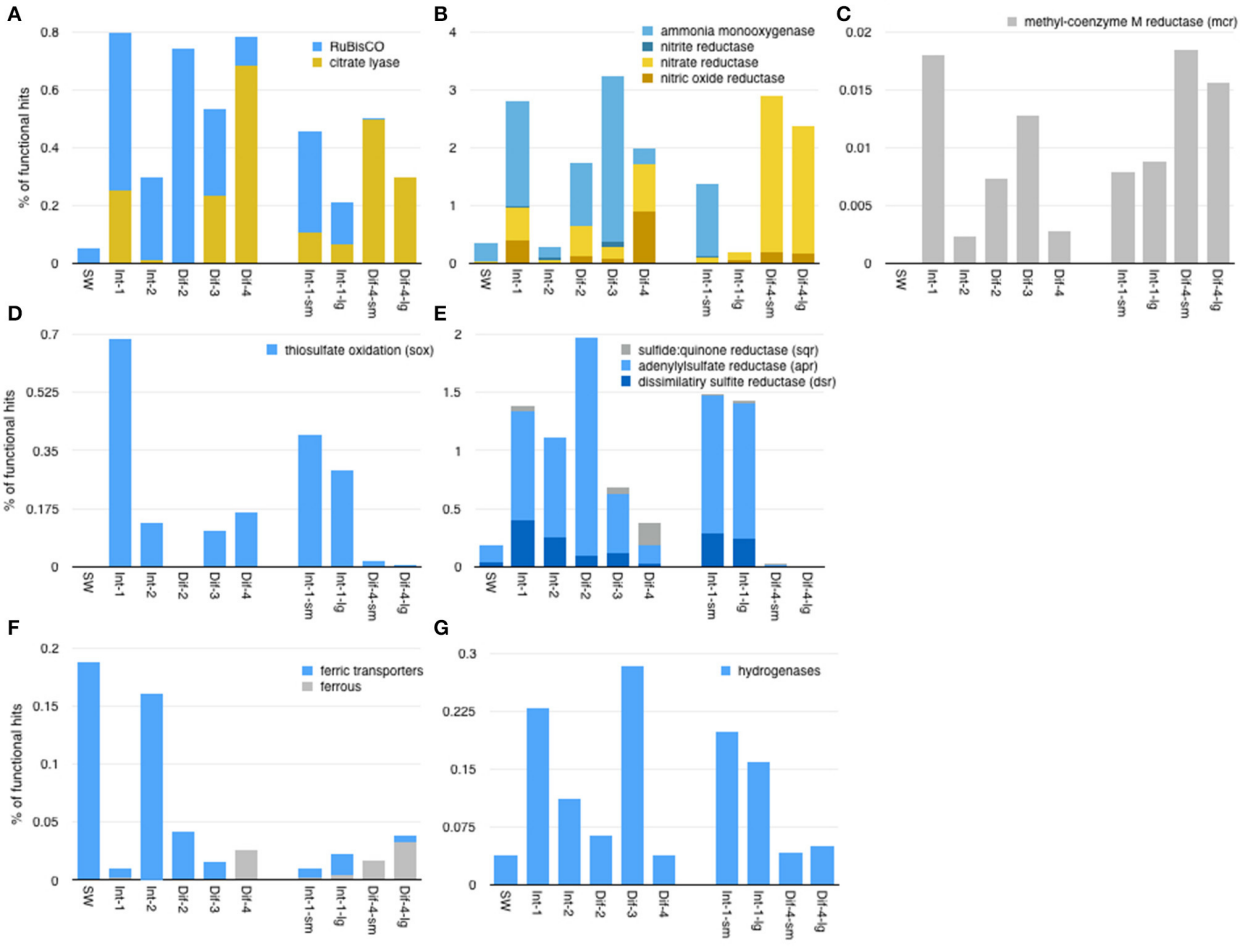
Expression of selected functional genes across the whole dataset related to **(A)** carbon fixation, **(B)** nitrogen metabolisms, **(C)** methanogenesis, **(D)** sulfur oxidation, **(E)** other sulfur metabolisms, **(F)** iron, and **(G)** hydrogen. Of these genes, only ammonia monooxygenase showed statistically significant differences in expression between sampling methods (higher expression in Niskin samples). Genes shown in blue showed statistically significantly more expression in Sites 1–4, while those in yellow were statistically significantly more expressed in Site 4 samples.

We were interested in examining the expression patterns of key genes related to chemosynthetic metabolisms and related to elemental cycling of carbon, nitrogen, sulfur, and iron, and hydrogen (Figure 8). The relative expression of genes allied to different carbon fixation pathways varied among the samples (Figure 8A). Total RuBisCO expression, indicative of the Calvin Benson Bassham (CBB) cycle, was highest at Site 2 and lowest at Site 4. In all samples, cbbM (representing RuBisCO form II was more highly expressed than cbbL (representing RuBisCO form I) which showed little to no expression (see Supplemental Table 4 for details). ATP citrate lyase, indicative of the reductive Tricarboxylic Acid (rTCA) Cycle, was most highly expressed at Site 4. Both of these carbon fixation genes were least expressed in the seawater sample, and both showed statistically significant differences in expression between Site 4 and Sites 1–3 (see Supplemental Table 3 for *p*-values).

With respect to nitrogen metabolism, gene expression involved in denitrification was highest in the Site 4 samples (Figure 8B). Nitrate and nitrite reductases were expressed in all samples, were predominantly dissimilatory (Supplemental Figures 3, 4), and had statistically significant differences in expression between Sites 1–3 and Site 4. Nitrate reductases were more highly expressed in Site 4, while nitrite reductases were more highly expressed in Sites 1–3. However, overall, nitrate reductase expression was far higher than that of nitrite reductases. Nitric oxide reductases followed a similar pattern as nitrate reductases, but nitrous oxide reductases were not well represented in these data (not shown). Ammonia monooxygenases showed a pattern opposite that of nitrate reductases, being both highly expressed and significantly more expressed in Sites 1–3 than Site 4.

Sulfur metabolism genes were expressed in all samples, but were least expressed in the seawater sample and Site 4 D-ESP samples (Figures 8D,E). The sulfur oxidation system (including soxABCXYZ), dissimilatory sulfite reductase (dsr genes including dsrMKOP, though not transcripts identified as assimilatory sulfite reductase), and adenylylsulfate reductase (including aprAB) all showed significantly higher expression in Sites 1–3 than Site 4.

Ferric iron transporters showed significantly more expression in Sites 1–3 than Site 4 (Figure 8F). Genes related to ferrous iron, on the other hand, showed higher expression in Site 4, but the difference was not statistically significant. The relative expression of hydrogenases (genes used to oxidize or detect hydrogen) were also significantly higher in Sites 1–3 than Site 4 (Figure 8G).

### Differential expression analysis of D-ESP samples

Differential expression analysis on selected samples (the three D-ESP samples with paired 0.2–5 and >5 μm size fractions, one from Site 1 and two from Site 4) highlighted transcripts that were differentially expressed between samples collected *in situ* by the D-ESP at Site 1 (intra-field) and Site 4 (the most hydrothermally influenced diffuse flow). **Figure 10** shows the most abundant differentially-expressed transcripts, plotting relative abundance and log odds ratio of those samples where average Site 1 and Site 4 relative abundance sum to >0.5%. Adenylylsulfate reductase (aprA, which encodes a key enzyme in microbial sulfate and sulfur oxidation), and particulate methane monooxygenase (pMMO) are among those transcripts most over-expressed in our Site 1 D-ESP samples relative to those from Site 4. In contrast, thiosulfate reductase, flagellin and flagellar proteins, nitrate and nitrite reductases, and hydrolase are among the most over-expressed in D-ESP Site 4 samples relative to those from Site 1.

## Discussion

The overarching goal of this effort was to determine the phylogenetic and functional changes of active microbial communities associated with diffuse-flow vent fluids as they disperse into the environment. Our hypothesis was that microbial activity (for which we used mRNA expression as a proxy) would track fluid geochemistry in a predictable manner. The results of this study, however, do not support the idea that microbial activity can be inferred from the fluid geochemistry. Instead, this work highlights the substantial heterogeneity of these systems and suggests that intra-field areas are a previously overlooked, high-productivity habitat.

Previous studies of low temperature, hydrothermally-influenced environments have focused largely on understanding the diversity and distribution of microbial taxa (Huber et al., 2002; Opatkiewicz et al., 2009; Anderson et al., 2013; Campbell et al., 2013; Meyer et al., 2013; Perner et al., 2013b). Other studies have focused on physiological capacities of target genotypes (e.g., the *Epsilonproteobacteria* in (Akerman et al., 2013); H_2_ oxidizers in (Perner et al., 2010); or denitrifying bacteria in Bourbonnais et al., 2012). However, the extent to which both seawater- and vent-derived microbes are active in these regions, as well as the extent to which geochemistry influences or indicates microbial processes, remain poorly constrained. In that regard, it has also been shown that community composition (as determined by metagenomics or 16S rDNA sequencing) is not an especially robust indicator of activity. Specifically, striking differences were found in the relative representation of Gamma- and *Epsilonproteobacteria* sequences recovered in DNA and RNA libraries constructed from ASHES diffuse flow fluids (Akerman et al., 2013). Our finding that the intra-field fluid among these diffuse vents was nearly indistinguishable, in terms of microbial activity, from some diffuse flows is of fundamental importance for any modeling efforts (discussed in Huber and Holden, 2008), as well as for predicting the global influence of hydrothermal vent microbial metabolism on geochemistry.

### Influence of geochemistry on the active microbial communities

In order to compare patterns of geochemistry and gene expression, we sampled three distinct habitats: non-vent seawater; intra-field fluid; and diffuse flow. All of the vent field microbial communities sampled here, both from diffuse flows or in intra-field fluid, were functionally distinct from our background seawater sample (Figure 8), indicating that the influence of diffuse hydrothermal venting extends beyond the obvious flows and potentially across the whole vent field. However, all of the chemical measurements we made did not show clear-cut distinctions between background seawater and vent field (diffuse and intra-field) fluids. Seawater and intra-field samples were geochemically similar in terms of pH, Si, and Mn concentrations, as well as the absence of detectable sulfide.

There was, however, vent signal throughout the vent field. Methane concentrations were higher in the intra-field than is typically found in background seawater (commonly single digit nM), which may explain some of the differences observed in gene expression. Surprisingly though, methane concentration and pH from Site 2 and 3 diffuse samples were more similar to intra-field samples than they were to Site 4 diffuse flows. Gene transcription did not appear to be predicted by geochemistry. The active communities from diffuse flow Site 4 were distinct from those of the diffuse flows at Sites 1–3 (Figures 4D, 5) despite the fact that Site 3 had detectable sulfide and elevated methane concentrations. Collectively, these expression and geochemistry data indicate that the intra-field fluid habitat is more similar to non-buoyant plumes typically associated with focused, higher temperature chimneys than it is to the background seawater.

It is often assumed that microbial activity is largely controlled by local geochemistry, and well-documented correlations between chemistry and microbiology support that generalization (e.g., Nakagawa et al., 2005; Huber et al., 2006; Perner et al., 2011 and references within; Flores et al., 2011; Akerman et al., 2013; Anderson et al., 2013; Anantharaman et al., 2015). However, our observations paint a picture that is more complicated than a simple correlation, indicating that much of vent microbial activity is not solely determined by the geochemical parameters measured here.

### Two distinct active vent field communities imply ecological niches that cross-cut canonical habitat boundaries

The active community at Site 4 was dominated by *Epsilonproteobacteria*, which are typically associated with hydrothermal vent diffuse flows (Nakagawa et al., 2005; Huber et al., 2007; Akerman et al., 2013; Anderson et al., 2013; Meyer et al., 2013). At the cooler diffuse Sites 2 and 3, where we might have predicted a proportional contribution of transcripts from vent-associated microbes, fluids were dominated by marine Gammaproteobacterial sulfur oxidizers (such as SUP05) commonly associated with hydrothermal buoyant plumes (Mattes et al., 2013) and anoxic waters (Wright et al., 2012). These organisms are typically found in low abundances in deep seawater, but are thought to become active in hydrothermal plumes (Sunamura et al., 2004; Dick and Tebo, 2010; Lesniewski et al., 2012; Anantharaman et al., 2013, 2015; Anderson et al., 2013; Sheik et al., 2015). Moreover, these diffuse flow communities were significantly more similar to those found at intra-field Sites 1 and 2, despite the differences in geochemistry (for *p*-values see Supplemental Table 3). Microbial activity at Sites 1–3 (both diffuse and intra-field samples) was, therefore, dominated by SUP05-like *Gammaproteobacteria* (taxa identified as SUP05 cluster or gammaproteobacterial vent symbionts). This is similar to the microbial community identified in a post-eruption “Snowblower” vent sampled shortly after our samples were collected (Meyer et al., 2013).

Our Site 4 *Epsilonproteobacteria*-dominated diffuse flow appears to have had less hydrothermal influence than other ASHES diffuse flows that have been shown to host more canonically vent-like activity profiles. One such vent (9 m) had a *Gammaproteobacteria*-dominated activity profile with pH 4.8 and average temperature of 49.7°C (Akerman et al., 2013). A recently published metatranscriptome of a diffuse flow at Marker 113 within the Axial caldera outside the ASHES vent field had comparable pH (6.2) to our Site 4 diffuse flow and lower temperature (24.1°C), but had a diverse expression profile characterized by substantial expression of *Alphaproteobacteria* and *Methanococcus*, in addition to *Epsilonproteobacteria* (Fortunato and Huber, 2016). These examples further imply that factors other than geochemistry (such as fluid dynamics or biological interactions) may explain why Site 4 was dominated by vent *Epsilonproteobacteria*.

Overall, these patterns imply that the active microbial community does not, as one might predict from thermodynamic models, directly mirror geochemistry (at least in terms of chemical species measured in this work). It has previously been demonstrated that vents in close proximity can host significantly different communities despite geochemical similarities (e.g., Opatkiewicz et al., 2009; Perner et al., 2013a,b; Sheik et al., 2015). It is important to note that we do not rule out all geochemical factors as determinants of community activity; for example, microbial energy metabolism in plumes may be determined in part by the nuances of plume-specific chemistry, such as sulfur availability (Anantharaman et al., 2015). It is also possible that additional measurements are needed to fully capture temporal variability in diffuse flow chemistry, and that such variability could be influencing microbial activity.

Oxygen concentration is one key factor that could be responsible for differences in overall microbial activity. Fortunato and Huber (2016) characterized an *in situ*-preserved, active community from a diffuse-flow vent at a different site (Marker 113) within Axial caldera, and interestingly showed a strikingly different active community far less dominated by either Gamma- or *Epsilonproteobacteria* than our diffuse flow metatranscriptomes. While oxygen was not quantified in their work, oxygen concentration, which is higher in cold background seawater and lower to absent in hydrothermal fluids, could explain this difference if we assume temperature is a proxy for oxygen concentration. Our Site 4 was 34.2°C, whereas the Marker 113 metatranscriptome was 24.1°C. However, it is impossible to rule out the effect of temperature itself on some other aspect of geochemistry (despite being lower temperature, Marker 113 had concentrations of methane, hydrogen sulfide, magnesium, and silica that were indicative of a greater hydrothermal influence than our Site 4 flow). Additionally, non-geochemical factors such as spatial variation between vent fields (Butterfield and Merle, 2007), or simply the substantial heterogeneity inherent in microbial activity, are also likely to influence community activity. Further research is needed to determine the relative influence of these factors.

### Functional gene expression patterns at sites 1–3: a ubiquitous seawater-derived *Gammaproteobacterial* community

Two of the most highly expressed genes in Sites 1–3 are versions of soluble methane monooxygenase (EC 1.14.13.25). In contrast, these genes were not detected in seawater and showed much lower expression in the Site 4 diffuse flow samples. This implies the community at Sites 1–3 is actively oxidizing methane for energy generation.

Sulfite reductase alpha subunit (EC 1.8.1.2, formerly 1.8.99.1) and adenylyl-sulfate reductase (aka APS reductase—aprA & aprB, EC 1.8.99.2) were also highly expressed in Sites 1–3 (ranking 13th and 14th most highly expressed, respectively), not expressed in the seawater sample, and had a much lower expression in Site 4 (ranking 284th and 166th, respectively). Both of these genes catalyze reversible reactions that can be part of either sulfate reduction or sulfide oxidation. Sulfite reductase works between sulfate to sulfide, and APS reductase between sulfite and adenylyl sulfate. From its expression alone, one cannot definitively determine whether it is playing a role in sulfate reduction or sulfide oxidation. However, given that Sites 1–3 are oxic and therefore not conducive to sulfate reduction, it seems likely that expression of these genes is indicative of sulfur oxidation. Additionally, the vast majority of sequences identified as APS reductase and sulfite reductase were most similar to sequences from Gammaproteobacterial symbionts, *Thiobacillus denitrificans*, and *Thioalkalivibrio* sp., most of which are known to perform sulfur oxidation. Because sulfide was not detected at Sites 1 and 2 and was relatively low at Site 3 (detection limit: ~1 uM), we suggest this habitat may support oxidation of partially reduced sulfur species such as thiosulfate, or elemental sulfur. Alternatively, this absence of sulfide may be due to active sulfide oxidation keeping concentrations below detection levels.

Expression of selected genes of interest further indicates that sulfur species oxidation appears to be an important aspect of metabolism throughout the vent field. While this has long been known to be true in vent environments, these data highlight the potential importance of these metabolic processes in the intra-field habitat. Dissimilatory sulfate reductase (*dsr*) genes are used both by anaerobic sulfate reducers and microaerophilic or aerobic sulfur oxidizers for sulfate reduction and sulfur oxidation, respectively. *dsr* genes were more highly expressed in the more oxic sites (1–3), and we infer that they reflect the prevalence of reduced sulfur compound oxidation. Other sulfur oxidation genes (*sox* and *apr*) showed similar expression patterns, further supporting the supposition that this process may be more pronounced in Sites 1–3 (Figures 8D,E). This activity profile is similar to those observed in a proteomic-based study of ASHES hydrothermal plumes sampled roughly 2 months after our samples were collected (Mattes et al., 2013). While we saw less expression of genes related to denitrification in Sites 1–3 than Site 4, there is evidence that many vent *Gammaproteobacteria* are also actively carrying out nitrate reduction. *Marinobacter*, a genus known to reduce nitrate (Pérez-Rodríguez et al., 2013), was particularly active in these sites. The lack of expression data in these sites may have more to do with underrepresentation of functional genes in the databases than an absence of denitrification.

Patterns of gene expression associated with carbon fixation indicate that primary productivity in Sites 1–3 is likely dominated by the CBB cycle (Figure 8A). Different carbon fixation pathways are predicted to dominate in different environments primarily because of the oxygen tolerance of key enzymes, and the CBB cycle is thought to be more favorable wherever oxygen is abundant (Hügler and Sievert, 2011). Because its concentration was lower in Site 4 than Site 1, it is plausible that oxygen was the primary driver for the observed patterns. Oxygen has been implicated in defining ecological niches for other marine microbes, such as marine ammonia-oxidizing archaea (e.g., Erguder et al., 2009), anaerobic and microaerophilic sulfate-reducing bacteria (reviewed in Baumgartner et al., 2006), and even soil microbial communities (e.g., Pett-Ridge and Firestone, 2005). It is likely that the ubiquity of CBB genes relates to the relatively higher oxygen concentrations in the field. Thus, it appears that, similar to the buoyant plumes from high temperature chimneys, intra-field environments are areas of increased primary productivity and therefore likely an ecologically important deep sea environment. However, it should also be noted that other factors co-vary with oxygen, including sulfide, temperature, and pH, and further work is needed to determine the extent to which these factor(s) govern the observed patterns.

### Activity profile at site 4: a vent-derived, *Epsilonproteobacteria*-dominated community

Sulfur oxidation also appeared to predominate at Site 4, though it is mediated by different biochemical pathways. Relative to Sites 1–3, sulfide:quinone reductase (*sqr*) was more highly expressed (though the differences were not statistically significant), and *dsr* genes were less highly expressed. This is consistent with sulfide oxidation to elemental sulfur, and similar to patterns of gene expression found in other low temperature vent environments where transcription from Gammaproteobacterial and Epsilonproteobacterial symbionts were shown to express different profiles of genes for sulfur metabolism (Yamamoto and Takai, 2011; Sanders et al., 2013). Notably, we also found elevated expression of genes associated with denitrification at Site 4 (Figure 8B). Denitrification is favored in lower oxygen regimes, and these data are consistent with the lower oxygen concentrations as well as the elevated hydrothermal contribution to chemistry at this location in conjunction with nitrate from seawater. Organisms similar to *Sulfurimonas denitrificans* and *Sulfurovum* sp. NBC37-1 (the most abundant species-level identifications at Site 4) dominated the overall active community at Site 4 and are known to be engaged in sulfide oxidation, hydrogen oxidation, and denitrification (Hoor, 1975; Nakagawa et al., 2007; Sievert et al., 2008). Furthermore, there is genetic evidence that nitrate reduction is an important metabolism in many *Epsilonproteobacteria* found at hydrothermal vents (Vetriani et al., 2014).

Based on ATP citrate lyase expression, carbon fixation at Site 4 may be dominated by the rTCA cycle. RuBisCO expression was also detected, though ~7- and 100-fold lower in abundance in Niskin and D-ESP samples, respectively (Figure 8A). The rTCA cycle is carried out by enzymes with higher oxygen sensitivity and is thought to dominate chemically-reduced hydrothermal vent environments (e.g., Campbell and Cary, 2004, and reviewed in Hügler and Sievert, 2011). Accordingly, we posit that denitrification is linked to sulfide oxidation—and ultimately carbon fixation—at Site 4.

### Differential expression analysis of paired D-ESP samples

**Figure 9:**
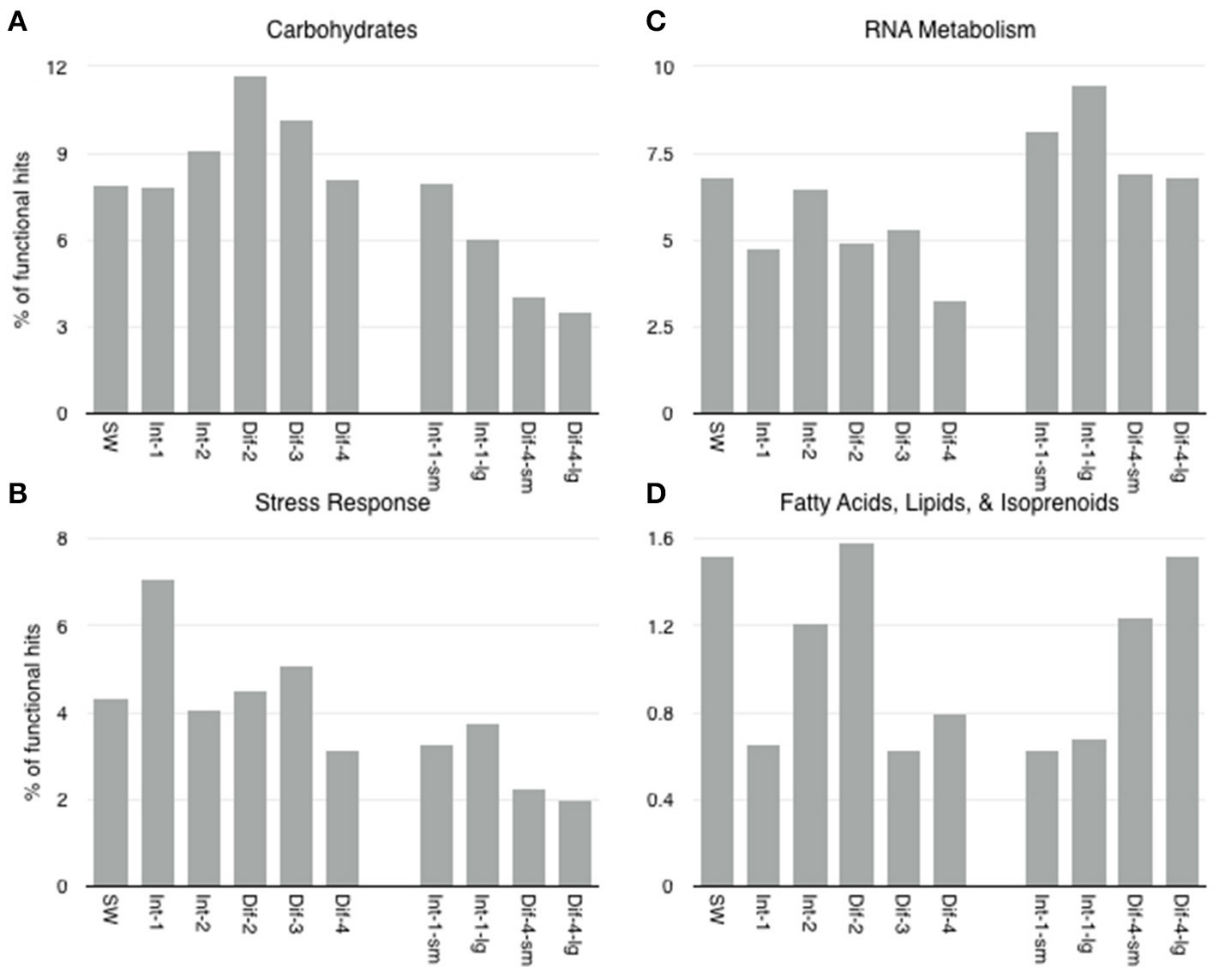
Level 1 SEED subsystems with expression that showed statistically significant difference in expression between sampling methods (Niskin vs. D-ESP) were **(A)** Carbohydrates, **(B)** Stress Response, **(C)** RNA Metabolism, and **(D)** Fatty Acids, Lipids, and Isoprenoids.

**Figure 10:**
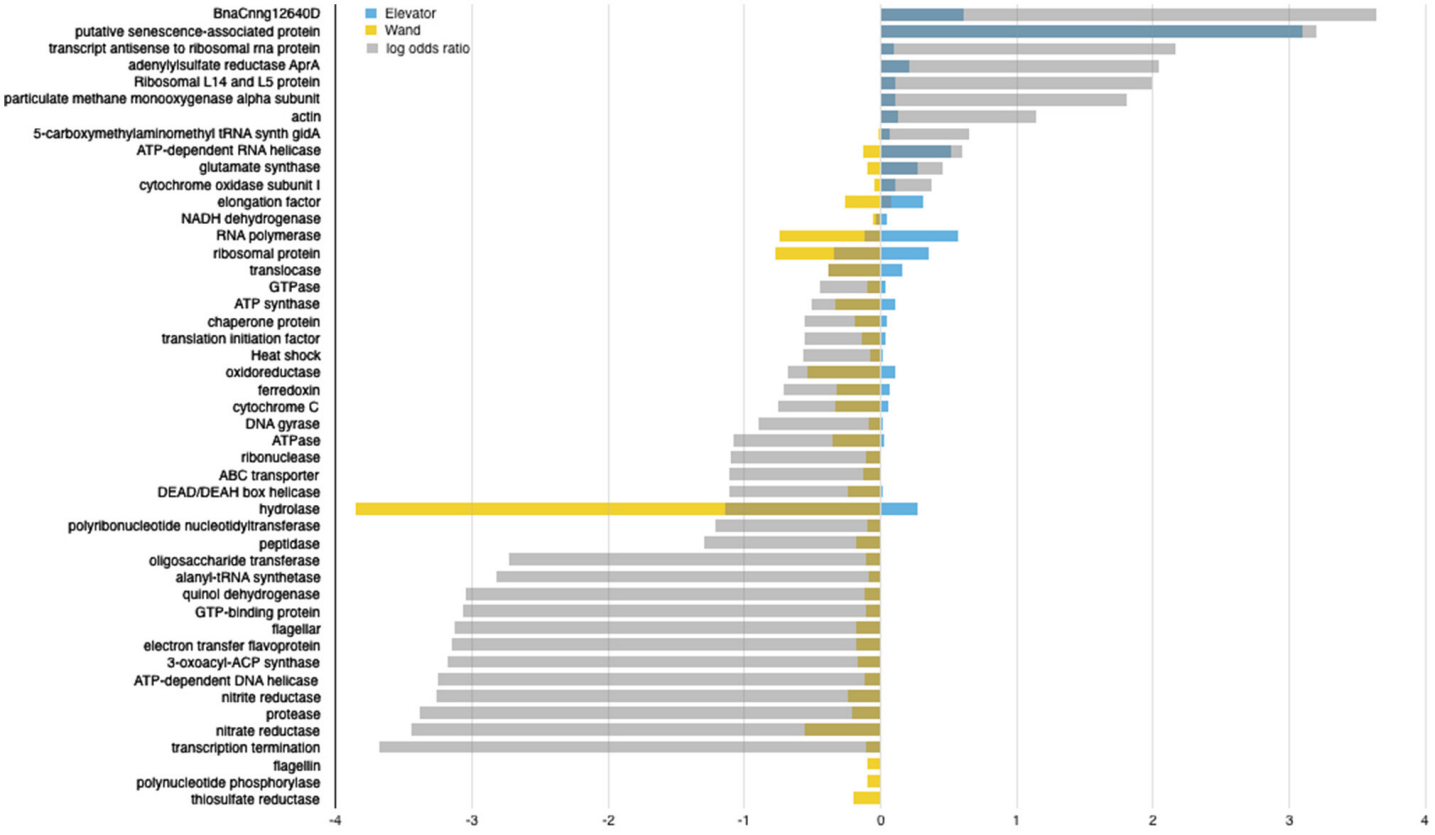
Differential expression analysis of D-ESP samples with sequenced filters of both 0.2–5 μM and >5 μM. Shown here are functional assignments with the highest log odds ratio or without log odds ratio due to expression in only the D-ESP wand (Site 4) or elevator (Site 1) samples. Log odds ratio between the two sites is shown in gray. Yellow and Blue bars represent Site 4 (wand) and Site 1 (elevator) expression, respectively.

To minimize any bias resulting from the two different sampling methods (Figure 9 shows functional categories showing significant differences by method, Figure 4B shows that diversity did not differ significantly between methods), we analyzed a subset of the D-ESP samples to quantitatively examine differences in gene expression between intra-field and diffuse flow fluids. Paired 0.2–5.0 μm and >5.0 μm D-ESP samples from the D-ESP elevator (Site 1) and D-ESP wand (Site 4) were used for this analysis (see Section Materials and Methods for details). Figure 10 shows the most highly expressed and significantly different transcript profiles between these two environments. A discussion of observed differences between sampling methods is available in the Supplemental Materials Results.

Gene expression patterns from the D-ESP elevator samples (intra-field Site 1) are more similar to the patterns observed in the other intra-field samples (i.e., those sampled via Niskin bottles), including *aprA* (one of the targeted genes showing a similar profile across the whole dataset, Figure 8), and a particulate methane monooxygenase (one of the most highly expressed genes in Sites 1–3, Supplemental Figures 3, 5).

Gene expression patterns in the D-ESP wand samples (Diffuse Site 4) are markedly different and seem to reflect a higher degree of heterogeneity in sulfur-based energy metabolism. Increased thiosulfate reductase expression potentially indicates thiosulfate oxidation, possibly coupled to nitrate reduction. Increased expression of nitrate and nitrite reductases (Figure 10, similar to the pattern of expression across the whole dataset—Figure 8) is also consistent with a lower oxygen environment. Increased expression of flagellin and other flagellar genes could potentially indicate increased chemotaxis and motility in diffuse flows relative to intra-field fluids.

## Conclusions

At the outset of this work, we asked whether microbial activity varies systematically among geochemically distinct environments. Based on the data presented herein, we posit that intra-field water (the fluids within the vent field without any visible signs of vent influence) is not comparable to background seawater, but rather appears to be a diffuse flow-derived non-buoyant plume, the physical extent of which remains unknown. Geochemical and gene expression data presented here are consistent with what has been observed in studies of buoyant plumes emitted from high temperature hydrothermal flows (Dick and Tebo, 2010; Lesniewski et al., 2012; Anantharaman et al., 2015). However, the data presented here are from cooler diffuse flows, and to our knowledge represent a microbial habitat that has not previously been identified or characterized. We also found the majority of activity in intra-field habitats likely came from seawater-derived microbes, while the communities derived from the warmer “core” of diffuse flows, such as Site 4, appear to be dominated by mesophilic and thermophilic microbes potentially derived from the subsurface. Additional microbiological data collected by the D-ESP but not discussed in this paper provide additional support for these two distinct active communities (see Supplemental Materials: Methods, Figure 5, and Tables 5, 6).

Deep sea hydrothermal vents and their plumes are known to have a substantial impact on global ocean chemistry (e.g., Resing et al., 2015). The global mid-ocean ridge system cycles the entire ocean volume-equivalent every 70,000–200,000 years (Johnson and Pruis, 2003; Wheat et al., 2003). This cycling is an important contribution to deep ocean iron and copper concentrations (Sander and Koschinsky, 2011), has influenced the global sulfur cycle over geologic time (reviewed in Bottrell and Newton, 2006), and likely plays an indirect, but important, role in governing total carbon export in certain locations of the ocean (Tagliabue et al., 2010). Given that the volume of fluids emitted by diffuse flow vents is comparable to or greater than that coming out of high temperature vents (Rona and Trivett, 1992; Baker et al., 1993; Bemis et al., 1993; Elderfield and Schultz, 1996; Wankel et al., 2011), it begs the question: How widespread are non-buoyant vent-field scale plumes, and how much of the Axial caldera is enveloped by such a plume? Future investigations should aim to further characterize the microbial communities in the intra-field fluids. That said, the data herein reveal striking differences in microbial gene expression among our diffuse flow sites, indicating that realized niches crosscut our traditional definitions of a vent habitat. Furthermore, analysis of intra-field samples highlights the extent to which microbes associated with hydrothermal systems—from warm diffuse flows to highly oxic intra-field fluids—are active beyond the confines of the easily distinguishable vent features.

## Author contributions

HO analyzed the data and wrote the manuscript with input from co-authors. PG and HO planned the work and collected and processed the Niskin samples. DR carried out the RNA extractions. CP designed the molecular assays for the D-ESP. WU carried out methane measurements. CS, JB, CP, WU, DP, SJ, and BR built, maintained and operated the D-ESP. MH carried out RuBisCO bioinformatic analyses.

### Conflict of interest statement

The authors declare that the research was conducted in the absence of any commercial or financial relationships that could be construed as a potential conflict of interest.
