# Supplementary material for: Co-registered Geochemistry and Metatranscriptomics Reveal Unexpected Distributions of Microbial Activity within a Hydrothermal Vent Field
Source: Front Microbiol. 2017 Jun 13;8:1042. doi: 10.3389/fmicb.2017.01042 (PMC5468400; doi:10.3389/fmicb.2017.01042)
Supplement: Supplementary file 1 [file Table1.DOCX]

**Supplemental Table 1**

| **D-ESP Sample Number** | **Corresponding MG-RAST ID** | **Sample Start time** | **Sample End Time** | **Int-1*** | **Dif-4*** |
| --- | --- | --- | --- | --- | --- |
| 1 | 4565284.3 | 7/13/11 19:09 | 7/13/11 19:25 | * |  |
| 7 | 4565472.3 | 7/14/11 16:29 | 7/14/11 16:46 | * |  |
| 23 | 4565473.3 | 7/17/11 1:08 | 7/17/11 1:23 |  | * |
| 29 | 4565474.3 | 7/18/11 0:34 | 7/18/11 0:49 |  | * |
| 30 | 4565475.3 | 7/18/11 3:24 | 7/18/11 3:39 |  | * |
| 31 | 4565464.3 | 7/18/11 6:12 | 7/18/11 6:26 |  | * |
