# Supplementary material for: Co-registered Geochemistry and Metatranscriptomics Reveal Unexpected Distributions of Microbial Activity within a Hydrothermal Vent Field
Source: Front Microbiol. 2017 Jun 13;8:1042. doi: 10.3389/fmicb.2017.01042 (PMC5468400; doi:10.3389/fmicb.2017.01042)
Supplement: Supplementary file 2 [file Table2.DOCX]

**Supplemental Table 2**

| **Description** | **Sample name** | **MG-RAST ID** | **Upload: sequence count** | **Post QC: sequence count** | **Post QC: mean sequence length** | **Post QC: mean GC percent** | **total M5RNA tax. IDs** | **total SEED functional IDs** | **Subsystem L1 IDs** |
| --- | --- | --- | --- | --- | --- | --- | --- | --- | --- |
| SW-Niskin | 3060 | 4551453.3 | 9,885,039 | 9,461,486 | 106 ± 26 | 51 ± 5 | 7,120,287 | 1,039,471 | 98,909 |
| Int-1-Niskin | 3007 | 4565465.3 | 15,015,234 | 14,526,420 | 112 ± 29 | 49 ± 5 | 16,593,713 | 1,922,098 | 212,005 |
| Int-1-Niskin | 3014 | 4565466.3 | 14,755,760 | 14,273,334 | 109 ± 28 | 48 ± 5 | 15,256,011 | 1,556,273 | 68,667 |
| Int-2-Niskin | 3237 | 4565468.3 | 17,876,264 | 17,235,158 | 110 ± 28 | 49 ± 5 | 20,986,145 | 2,470,522 | 453,647 |
| Int-2-Niskin | 3238 | 4565469.3 | 15,855,175 | 15,308,541 | 109 ± 28 | 50 ± 5 | 18,283,775 | 2,117,804 | 439,818 |
| Int-2-Niskin | 3239 | 4565470.3 | 15,605,206 | 15,077,545 | 111 ± 29 | 50 ± 5 | 18,299,932 | 2,239,360 | 413,146 |
| Dif-2-Niskin | 3234 | 4551455.3 | 10,887,331 | 10,435,245 | 107 ± 26 | 50 ± 5 | 8,287,205 | 1,292,912 | 107,999 |
| Dif-3-Niskin | 3495 | 4551443.3 | 10,368,683 | 9,968,436 | 115 ± 30 | 50 ± 5 | 7,775,902 | 1,114,581 | 93,698 |
| Dif-3-Niskin | 3494 | 4565467.3 | 21,472,651 | 20,759,160 | 109 ± 27 | 49 ± 5 | 22,264,145 | 2,244,846 | 152,401 |
| Dif-4-Niskin | 3471 | 4565471.3 | 16,567,419 | 16,074,027 | 112 ± 29 | 48 ± 5 | 17,395,616 | 2,449,093 | 358,446 |
| Dif-4b-Niskin | 3498 | 4551454.3 | 13,494,976 | 13,034,080 | 108 ± 27 | 48 ± 5 | 8,590,917 | 1,287,766 | 83,800 |
| Int-1-lg-ESP | WCR 1 | 4620934.3 | 14,057,110 | 13,664,800 | 112 ± 29 | 46 ± 7 | 9,529,438 | 1,142,981 | 62,739 |
| Int-1-sm-ESP | WCR 1 | 4565284.3 | 14,565,731 | 14,086,549 | 113 ± 30 | 50 ± 5 | 16,196,605 | 1,860,167 | 183,080 |
| Int-1-sm-ESP | WCR 7 | 4565472.3 | 10,660,730 | 10,127,396 | 103 ± 21 | 49 ± 5 | 8,397,646 | 1,147,626 | 44,080 |
| Dif-4-sm-ESP | WCR 23 | 4565473.3 | 14,053,708 | 13,609,944 | 109 ± 27 | 48 ± 5 | 9,464,190 | 1,413,252 | 313,728 |
| Dif-4-lg-ESP | WCR 23 | 4620935.3 | 15,849,209 | 15,379,195 | 109 ± 27 | 48 ± 5 | 11,228,550 | 2,198,898 | 377,913 |
| Dif-4-sm-ESP | WCR 29 | 4565474.3 | 19,944,458 | 19,313,650 | 104 ± 23 | 48 ± 5 | 18,516,154 | 1,906,272 | 336,112 |
| Dif-4-sm-ESP | WCR 30 | 4565475.3 | 16,013,080 | 15,518,719 | 109 ± 27 | 47 ± 6 | 14,340,278 | 1,708,959 | 554,398 |
| Dif-4-lg-ESP | WCR 31 | 4620933.3 | 14,888,091 | 14,452,517 | 109 ± 28 | 48 ± 6 | 10,497,031 | 2,261,309 | 519,431 |
| Dif-4-sm-ESP | WCR 31 | 4565464.3 | 15,453,393 | 14,995,631 | 109 ± 28 | 47 ± 6 | 13,392,976 | 1,751,585 | 644,363 |
