# Supplementary material for: Co-registered Geochemistry and Metatranscriptomics Reveal Unexpected Distributions of Microbial Activity within a Hydrothermal Vent Field
Source: Front Microbiol. 2017 Jun 13;8:1042. doi: 10.3389/fmicb.2017.01042 (PMC5468400; doi:10.3389/fmicb.2017.01042)
Supplement: Supplementary file 3 [file Table3.DOCX]

**Supplemental Table 3.**

| **Selected Functional Genes: Figure 8** | |  |  |  |
| --- | --- | --- | --- | --- |
|  | Kruskal | Wilcox | Wilcox | Wilcox |
| **graph label** | by site | by envi | by altenvi | by method |
| all Rubisco | **0.0092** | 0.0328 | **0.0002** | 0.0350 |
| all Citrate Lyase | **0.0028** | **0.0091** | **0.0003** | 0.2428 |
| all sox | **0.0208** | **0.0012** | **0.0121** | 0.3562 |
| all sqr | 0.1459 | 0.3817 | 0.5882 | 0.5367 |
| all apr | **0.0067** | **0.0068** | **0.0001** | 0.0653 |
| all dsr | **0.0041** | **0.0025** | **0.0001** | 0.0279 |
| all ammonia monooxygenase | **0.0059** | 0.1259 | **0.0014** | **0.0079** |
| all nitrite reductase | 0.0363 | 0.1266 | **0.0164** | 0.0521 |
| all nitrate reductase | **0.0069** | **0.0003** | **0.0002** | 0.0653 |
| all nitric oxide reductase | **0.0150** | **0.0050** | **0.0012** | 0.6038 |
| ferric transporters | 0.2411 | 0.7547 | 0.1290 | 0.1120 |
| ferrous | **0.0055** | 0.0826 | **0.0059** | 0.0491 |
| mcr | 0.3173 | 0.8362 | 0.9013 | 0.5396 |
| hydrogenases | **0.0095** | 0.0506 | **0.0008** | 0.1128 |
|  |  |  |  |  |
| # of tests | 56.0000 |  |  |  |
| bonferroni | 0.0009 | **highly sig** |  |  |
| Benjamini-Hochberg | 0.0254 | **sig** |  |  |
|  |  |  |  |  |
| **SEED L1 Subsystems: Figure 7** | |  |  |  |
| test | Kruskal | Wilcox | Wilcox | Wilcox |
|  | site | envi | altenvi | method |
| protein_metabolism | 0.4666 | 0.9039 | 0.2060 | 0.1128 |
| clustering_based_subsystems | 0.2559 | 0.7168 | 0.3511 | 0.1564 |
| respiration | 0.1512 | 0.5999 | 0.2060 | 0.1128 |
| carbohydrates | **0.0103** | 0.3511 | **0.0018** | **0.0010** |
| RNA_metabolism | 0.4493 | 0.2060 | 0.9678 | **0.0006** |
| amino_acids_and_derivatives | 0.0765 | 0.3950 | 0.7780 | 0.2775 |
| miscellaneous | 0.5779 | 0.7780 | 0.2723 | 0.0279 |
| cofactors_vitamins_prosthetic_groups_and_pigments | **0.0040** | **0.0025** | **0.0000** | 0.4002 |
| N_metabolism | **0.0053** | **0.0036** | **0.0001** | 0.1823 |
| S_metabolism | 0.1078 | 0.0620 | 0.0328 | 0.0789 |
| stress_response | **0.0091** | 0.0620 | **0.0003** | **0.0006** |
| phages_prophates_transposable_elements_and_plasmids | **0.0228** | 0.3100 | **0.0025** | **0.0101** |
| membrate_transport | **0.0154** | 0.1087 | **0.0018** | **0.0133** |
| nucleosides_and_nucleotides | **0.0224** | 0.0259 | **0.0018** | 0.0279 |
| cell_wall_and_capsule | **0.0248** | 0.0506 | 0.0908 | 0.4002 |
| DNA_metabolism | 0.4798 | 0.0754 | 0.0409 | 0.4002 |
| fatty_acids_lipids_and_isoprenoids | **0.0071** | 0.9039 | 0.1288 | **0.0000** |
| cell_division_and_cell_cycle | 0.0884 | 0.9039 | 0.1288 | 0.4002 |
| virulence_disease_and_defense | 0.0275 | 0.1518 | **0.0121** | 0.1823 |
| regulation_and_cell_signalling | 0.6638 | 0.5448 | 0.5448 | 0.1128 |
| motility_and_chemotaxis | 0.4042 | 0.3511 | 0.7780 | 0.0947 |
| P_metabolism | **0.0134** | **0.0018** | **0.0005** | 0.7197 |
| metabolism_of_aromatic_compounds | **0.0055** | 0.0754 | **0.0018** | 0.0947 |
| Fe_acquisition_and_metabolism | 0.0377 | 0.2304 | 0.1998 | 0.0936 |
| secondary_metabolism | **0.0031** | **0.0050** | **0.0000** | 0.3562 |
| K_metabolism | **0.0252** | 0.0746 | **0.0114** | 0.0927 |
| dormancy_and_sporulation | 0.2488 | 0.4759 | 0.3348 | 0.7716 |
| photosynthesis | 0.4747 | 0.2403 | 0.1311 | 0.0513 |
|  |  |  |  |  |
| # of tests | 112.0000 |  |  |  |
| bonferroni | 0.0004 | **highly sig** |  |  |
| Benjamini-Hochberg | 0.0252 | **sig** |  |  |
|  |  |  |  |  |
| **Taxonomic IDs: Figure 6** |  |  |  |  |
|  | Kruskal | Wilcox | Wilcox | Wilcox |
| **csv label** | by site | by envi | by altenvi | by method |
| Archaea | 0.0287 | 0.2723 | **0.0050** | **0.0004** |
| Eukaryota | **0.0032** | **0.0204** | **0.0001** | 0.0435 |
| Bacteria | **0.0047** | **0.0012** | **0.0001** | 0.0535 |
| other | **0.0052** | **0.0121** | **0.0003** | **0.0101** |
| Actinobacteria | **0.0038** | 0.0328 | **0.0001** | **0.0010** |
| Firmicutes | 0.0530 | 0.4920 | 0.0259 | **0.0220** |
| Bacteroidetes | **0.0031** | **0.0008** | **0.0000** | **0.0076** |
| unclassified bacteria | **0.0073** | **0.0204** | **0.0005** | 0.0435 |
| Proteobacteria | 0.1573 | 0.3100 | 0.1288 | 0.7802 |
| Zetaproteobacteria | **0.0198** | 0.1288 | **0.0025** | 0.0435 |
| Deltaproteobacteria | 0.0763 | 0.1774 | 0.0259 | 0.0350 |
| unclassified proteobacteria | **0.0038** | **0.0008** | **0.0000** | 0.2775 |
| Alphaproteobacteria | **0.0045** | **0.0018** | **0.0001** | 0.0653 |
| Betaproteobacteria | **0.0034** | **0.0204** | **0.0000** | 0.0535 |
| Epsilonproteobacteria | **0.0028** | **0.0003** | **0.0000** | 0.0535 |
| Gammaproteobacteria | **0.0042** | **0.0018** | **0.0000** | **0.0172** |
|  |  |  |  |  |
| # of tests | 64.0000 |  | 64.0000 |  |
| bonferroni | 0.0008 | **highly sig** |  |  |
| Benjamini-Hochberg | 0.0254 | **sig** |  |  |
