# Supplementary material for: Co-registered Geochemistry and Metatranscriptomics Reveal Unexpected Distributions of Microbial Activity within a Hydrothermal Vent Field
Source: Front Microbiol. 2017 Jun 13;8:1042. doi: 10.3389/fmicb.2017.01042 (PMC5468400; doi:10.3389/fmicb.2017.01042)
Supplement: Supplementary file 4 [file Table4.DOCX]

**Supplemental Table 4.**

| **Sample Type** | **MG-RAST ID** | **Counts (RPKM normalized counts)** | | **Hits** | |
| --- | --- | --- | --- | --- | --- |
|  |  | **cbbL** | **cbbM** | **cbbL** | **cbbM** |
| SW-Niskin | 3060 | 0 | 6.091340353 | 0 | 19 |
| Int-1-Niskin | 9800X12_re-do_20140523 | 2.115515167 | 118.2170023 | 3 | 559 |
| Int-1-Niskin | 9800X13_re-do_20140523 | 0 | 6.912729682 | 1 | 37 |
| Int-2-Niskin | 9800X15_re-do_20140523 | 1.224700669 | 72.91342914 | 2 | 349 |
| Int-2-Niskin | 9800X16_re-do_20140523 | 0 | 122.2069212 | 0 | 427 |
| Int-2-Niskin | 9800X17_re-do_20140523 | 2.429128774 | 96.9199572 | 3 | 319 |
| Dif-2-Niskin | 3234 | 12.63988801 | 92.51695808 | 12 | 256 |
| Dif-3-Niskin | 3495 | 1.876396156 | 39.27029098 | 2 | 113 |
| Dif-3-Niskin | 9800X14_re-do_20140523 | 5.288126666 | 37.07284568 | 9 | 138 |
| Dif-4-Niskin | 9800X18_re-do_20140523 | 1.986788361 | 0.321803749 | 4 | 2 |
| Dif-4b-Niskin | 3498 | 2.738560031 | 36.57238248 | 3 | 104 |
| Int-1-lg-ESP | 9800X4-re-do-20150302 | 0 | 13.12568391 | 0 | 54 |
| Int-1-sm-ESP | 9800X3-re-do-20140520 | 0 | 45.60933968 | 0 | 195 |
| Int-1-sm-ESP | 9800X5_re-do_20140523 | 0 | 48.51950343 | 0 | 118 |
| Dif-4-sm-ESP | 9800X6_re-do_20140523 | 0 | 0 | 0 | 4 |
| Dif-4-lg-ESP | 9800X7_re-do_20150302 | 0 | 1.092988999 | 0 | 4 |
| Dif-4-sm-ESP | 9800X8_re-do_20140523 | 0 | 1.025084757 | 0 | 7 |
| Dif-4-sm-ESP | 9800X9_re-do_20140523 | 0.698420348 | 1.746050869 | 1 | 18 |
| Dif-4-lg-ESP | 9800X11_re-do_20150302 | 0 | 1.2796793 | 0 | 6 |
| Dif-4-sm-ESP | 9800X10_re-do_20140523 | 0 | 7.965665847 | 0 | 20 |
