# Supplementary material for: Co-registered Geochemistry and Metatranscriptomics Reveal Unexpected Distributions of Microbial Activity within a Hydrothermal Vent Field
Source: Front Microbiol. 2017 Jun 13;8:1042. doi: 10.3389/fmicb.2017.01042 (PMC5468400; doi:10.3389/fmicb.2017.01042)
Supplement: Supplementary file 5 [file Table5.DOCX]

| **Supplemental Table 5.** | |  |  |  |  |  |
| --- | --- | --- | --- | --- | --- | --- |
| **Target Group** | **Probe Name** | **Probe Type** | **Sequence** | **Reference** | **M. aceotvorans Culture*** | **M. jannaschii Culture*** |
| Marine G1 crenarchaea | MarineCren_554 | capture | biotin-(C9)3-GATGCTTTAGGCCCAATAATCAMCCT | Preston et al 2009 | Positive | Positive |
| Marine G2 crenarchaea | MarineEuryII_547 | capture | biotin-(C9)3-TTAGGCCCAATAAAAKTGACKACCACT | Preston et al 2009 | ND | ND |
| Pelagibacter | SAR11_441 | capture | biotin-(C9)3-TACAGTCATTWTCTTCCCCKACGAAGAG | Preston et al 2009 | ND | ND |
| KTCC119 | KTC1119_441 | capture | biotin-(C9)3-CCGTGAACCTTTCCTCCCAATT | Preston et al 2009 | ND | ND |
| MAlph | Malph-1_488 | capture | biotin-(C9)3-GCCGGGGTTTCTTTACCA | Preston et al 2009 | ND | ND |
| ARCTICBD19-96 | ARCTIC96BD-19_443b | capture | TATTAACCGTTAACTTTTCTTCACAA-(C9)3-biotin | Preston et al 2009 | ND | ND |
| Marine Delta | DeltaEB7501B7_642 | capture | biotin-(C9)3-CTG**A**CCCTCTCTAGATTAGYAG | This Study | ND | ND |
| Methococcus | MethococcB | capture | biotin-(C9)3-GCACCGGACTTGCCCAGC | This Study | ND | Positive |
| Methanomicrobiales | MethMicro498 | capture | biotin-(C9)3-GGTCTTGCCCGGCCCTTTCT | This Study | Positive | Positive |
| Methanomicrobiales | MethMicro252 | capture | biotin-(C9)3-GGCTTGTTGGGCCGTTACC | This Study | ND | ND |
| Ridgeia symbiont | RidSym | capture | biotin-(C9)3-GACCCAAGGGTATTATCCTC | This Study | ND | ND |
| Methanosarcina | MethSar280 | capture | biotin-(C9)3-CTCTCACAACCCGTACCCGT | This Study | Positive | ND |
| *Preliminary tests were performed in a laboratory-based 96-well plate format as previously described (Greenfield et al 2008, Preston et al 2009) using cultures of M. jannashii and M. acetovorans. | | | | | | |
