# Supplementary material for: Co-registered Geochemistry and Metatranscriptomics Reveal Unexpected Distributions of Microbial Activity within a Hydrothermal Vent Field
Source: Front Microbiol. 2017 Jun 13;8:1042. doi: 10.3389/fmicb.2017.01042 (PMC5468400; doi:10.3389/fmicb.2017.01042)
Supplement: Supplementary file 6 [file Table6.DOCX]

**Supplemental Table 6**

| Assay | Forward (5' to 3') | Reverse | Probe | Slope | Intercept | % Efficiency | Reference |
| --- | --- | --- | --- | --- | --- | --- | --- |
| 16SrRNAThaumarchaeota | AGATGGGTACTGAGACACGGAC | CTGTAGGCCCAATAATCATCCT | FAM-TTACCGCGGCGGCTGGCAC-BHQ_1 | -3.269 | 38.700 | 102.24 | Suzuki et al., 2000 |
| WCB-amoA | CATCCRATGTGGATTCCATCDTG | AAYGCAGTTTCTAGYGGATC | FAM-CCAAAGAATATYAGCGARTG-BHQ_1 | -3.283 | 37.694 | 101.6 | Moiser et al., 2011 |
