# Supplementary material for: Co-registered Geochemistry and Metatranscriptomics Reveal Unexpected Distributions of Microbial Activity within a Hydrothermal Vent Field
Source: Front Microbiol. 2017 Jun 13;8:1042. doi: 10.3389/fmicb.2017.01042 (PMC5468400; doi:10.3389/fmicb.2017.01042)
Supplement: Supplementary file 7 [file DataSheet1.docx]

Supplementary Material

**Title**

Co-registered geochemistry and metatranscriptomics reveal unexpected distributions of microbial activity within a hydrothermal vent field

**Authors**: Heather Olins^1*^, Daniel Rogers^2^, Christina Preston^3^, William Ussler III^3^, Douglas Pargett^3^, Scott Jensen^3^, Brent Roman^3^, James Birch^3^, Christopher Scholin^3^, M. Fauzi Haroon^1^, and Peter Girguis^1^

^1^Harvard University, Department of Organismic and Evolutionary Biology

^2^Stonehill College, Department of Chemistry

^3^Monterey Bay Aquarium Research Institute (MBARI)

**Correspondence:**

Heather Olins

[heatherolins@gmail.com](mailto:heatherolins@gmail.com)

**
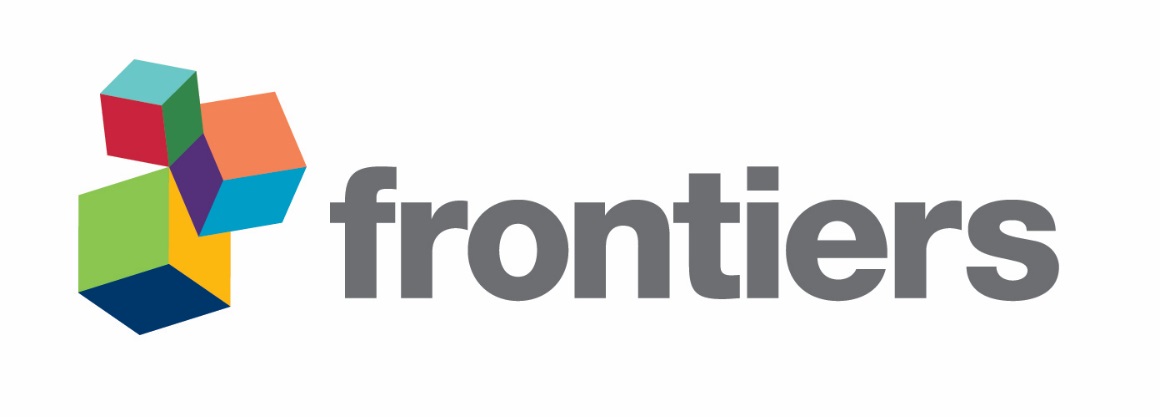
**

**Methods**

D-ESP sampling

For this and associated efforts, the D-ESP was fitted with an *in situ* mass spectrometer (ISMS; Wankel et al, 2011) for co-registered analyses of dissolved volatiles (data not shown). The ISMS and CTD shared a common in-let and were in series with ISMS first. Of the 5L samples collected, the decompressed sample was partitioned whereby 1L was archived in RNALater providing the samples used in this work. 2L went to real-time analyses using DNA probe arrays and quantitative PCR to detect a variety of ribosomal RNA and target gene sequences (data not presented in this work; Preston et al., 2009; Ussler et al., 2013).

Metatranscriptomic Sequencing

Library construction is performed using the Illumina TruSeq Stranded mRNA Sample NA Preparation Kit (cat# FC-122-2101, FC-122-2102 or FC-121-2103) using the following methods. Poly-A RNA is purified from total RNA (100 ng to 4 ug) using poly-T oligo-attached magnetic beads. The Poly-A RNA is eluted from the beads and fragmented with divalent cations under elevated temperature. RNA fragments are copied into first strand cDNA using random primers and Superscript II Reverse Transcriptase. Second strand cDNA synthesis is accomplished using DNA polymerase I and Rnase H under conditions in which dUTP is substituted for dTTP and yields blunt-ended cDNA in which the second strand is marked with dUTP. An A-base is added to the blunt ends as a means to prepare the cDNA fragments for adapter ligation and block concatamer formation during the ligation step. Adapters containing a T-base overhang are ligated to the A-tailed DNA fragments. Ligated fragments are PCR-amplified (12-15 cycles). PCR enables amplification of the first strand cDNA product, whereas attempted amplification of the second strand product stalls at dUTP bases and therefore is not enriched. Following amplification, the library is purified by bead based methodologies.

The concentration of the amplified library is measured using the Invitrogen Qubit dsDNA HS Assay (Q32851) and an aliquot of the library is resolved on an Agilent 2200 Tape Station using a D1K (cat# 5067-5361 and 5067-5362) or a High Sensitivity D1K (cat# 5067-5363 and 5067-5364) assay to define the size range. Libraries are adjusted to a concentration of approximately 10 nM and quantitative PCR is performed using the KapaBiosystems Kapa Library Quant Kit (cat# KK4824) to quantitate adapter ligated library molecules. The concentration is further adjusted following qPCR to prepare the library for Illumina sequence analysis.

RuBisCO forms

To further investigate the dominant Calvin-Benson Cycle form that was expressed in the samples, the two established marker genes cbbL (form I) and cbbM (form II) were quantified from the unassembled metatranscriptomic reads using ShortBRED (Kaminiski et al., 2015). Firstly, shortbred-identify was used to generate unique peptide markers from all cbbL (n=685) and cbbM (n=108) gene sequences in Integrated Microbial Genomes (IMG) (Markowitz et al., 2012; accessed on 28 Dec. 2016) using default values. Next, shortbred-quantify was performed to map the translated metatranscriptomic reads to the peptide markers at ≥95% amino acid identity across ≥95% of the marker length and values shown are in reads per kilobase per million reads (RPKM).

Additional D-ESP microbiology

Each time the deep water sampling module (DWSM) sampled seawater in the vent field to archive samples, a fraction of seawater was filtered, lysed and analyzed in situ by the 2G ESP-MFB using both sandwich hybridization (SHA) and quantitative PCR (qPCR) as previously described (Preston et al 2011, Ussler et al 2013). Briefly, particulates collected from 2L samples were lysed in 1.4ml of 3M GuSCN (pH 8.9, Preston et al., 2011) at 85°C for 8 minutes. Lysate was then filtered through a 0.22µm filter and used to run both SHA and qPCR.

For sandwich hybridization, 0.5 ml of the unpurified lysate was diluted 1:1 with lysis diluent and applied to arrays spotted with RNA-targeted, DNA capture probes. Several probes targeted microbial groups detected within the Juan de Fuca vent environment. (Supplementary Table 5). Detection of hybridization to capture probes was as previously described (Greenfield et al., 2008) using general signal probes that target bacteria and archaea (Preston et al., 2009). Array analysis software (<https://drive.google.com/file/d/0B8RHIr7J8XHoclQ3d0pUMU5Ua2c/view>) was used to determine probe spot intensity. Averaged signal intensities greater than average background plus three times the background standard deviation were considered positive.

DNA was extracted from a second aliquot of lysate and used in qPCR assays to quantify 16S rRNA and amoA group B genes from Thaumarchaeota (Supplementary Table 6) as previously described (Preston et al., 2011, Robidart et al 2011, Varljay et al 2015, Yamahara et al., 2015). The abundance of the gene per ml seawater was determined using standard curves using linearized plasmids of cloned PCR products. PCR efficiency of each assay (Supplementary Table 6) was similar to those run in the laboratory on standard equipment. In addition, little variation (<5%) was observed between the efficiency of pre- and post- deployment standard curves (data not shown).

**Results**

Sampling Methods Comparison

Technical limitations, including limited ship time and the associated challenges in deploying the D-ESP at multiple sites throughout the vent field, led us to collect samples via ROV-based Niskin bottles to supplement the D-ESP samples. As such, we were interested in how differences in sampling method influenced the resulting data, namely community composition and expression activity. Some level 1 subsystems showed statistically significant differences in expression between the two sampling methods (Figure 7). Two categories were more highly expressed in Niskin samples than D-ESP samples: Carbohydrates and Stress Response. The Carbohydrates category was dominated by increased expression of genes related to the Glyoxylate bypass, the TCA Cycle, the Calvin-Benson Cycle, and the Pentose Phosphate Pathway among others. The Stress Response category was dominated by expression of genes related to heat shock, cold shock, oxidative stress, and redox-dependent regulation of nucleus processes. RNA metabolism (a category that was dominated by expression of DNA-directed RNA polymerase) was expressed more highly in D-ESP samples. The final L1 subsystem that was showed significant difference of expression between sampling methods was Fatty Acids, Lipids, and Isoprenoids (Figure 7d).

Despite significant differences between the sampling methods overall, both methods showed the same pattern of expression differences between Sites 1-3 and Site 4 in the RNA Metabolism and Stress Response categories. In both of these cases Site 4 showed lower expression than Sites 1-3. In contrast, the carbohydrates and fatty acids, lipids, and isoprenoids did not show a consistent pattern between Sites 1-3 and Site 4.

Of the selected functional genes described in the previous section, the only one to have statistically significant difference in expression between the sampling methods was ammonia monooxygenase (Figure 8b). Ammonia monooxygenases were more highly expressed in Niskin samples than D-ESP samples, but both sampling methods showed higher expression in Sites 1-3 than Site 4. Of the taxonomic groupings, the only taxa to have statistically significant differences in representation between sampling methods was *Gammaproteobacteria* (Figure 6c). Like ammonia monooxygenase, *Gammaproteobacteria* reads were more abundant in the Niskin samples, but both sampling methods showed higher abundance in Sites 1-3 than Site 4.

**Supplemental Table Captions**

Supplemental Table 1

Subset of the D-ESP sampling schedule showing when and where the

D-ESP samples used in this work were collected during its multi-day deployment.

Supplemental Table 2

Details of the metatranscriptomic data sets uploaded to, and analyzed within MG-RAST.

Supplemental Table 3

P-values from statistical tests used in this work. Significant differences are highlighted in selected genes, L1 Seed Subststems, and taxa by site, environment, alternative environment (site 1-3 vs. site 4), or sampling method. These values correspond to Figures 8, 7, and 6 respectively from the main manuscript.

Supplemental Table 4

Data from RuBiscCO expression analysis showing counts and hits of Rubisco forms (cbbL vs. cbbM) in each metatranscriptome.

Supplemental Table 5

Details of probes used in D-ESP sandwich hybridization array (SHA). Data shown in Supplemental Figure 5.

Supplemental Table 6

Details of the probes used in the D-ESP qPCR assays. Data shown in Supplemental Figure 5.

**References**

Greenfield D, Marin III R, Doucette JG, Mikulski C, Jensen S, Roman B, et al. (2008). Field applications of the second-generation Environmental Sample Processor (ESP) for remote detection of harmful algae: 2006- 2007. *Limnology and Oceanography: Methods* 6: 667- 679.

Kaminski J, Gibson MK, Franzosa EA, Segata N, Dantas G, Huttenhower C (2015). High- Specificity Targeted Functional Profiling in Microbial Communities with ShortBRED. *PLoS Comput Biol* 11 (12).

Markowitz V, Chen I, Palaniappan K, Chu K, Szeto E, Grechkin Y, *et al.* (2012). IMG: the integrated microbial genomes database and comparative analysis system. *Nucl Acids Res* 40 (D1) D115-D122.

Mosier AC, Francis CA, (2011). Determining the distribution of marine and coastal ammonia-oxidizing archaea and bacteria using a quantitative approach. In: Martin GK (ed). Methods in Enzymology. Academic Press: New York, pp 205–221.

Preston C, Marin R III, Jenson S, Feldman J, Massion E, DeLong E, *et al.* (2009). Near real-time, autonomous detection of marine bacterioplankton on a coastal mooring in Monterey Bay, California, using rRNA-targeted DNA probes. *Environ Microbiol* B:1168-1180.

Preston CM, Harris A, Ryan JP, Roman B, Marin III R, Jensen S, et al., (2011). Underwater application of quantitative PCR on an ocean mooring.  *PLoS ONE*, 6(8): e22522.

Robidart JC, Preston CM, Paerl RW, Turk KA, Mosier AC, Francis CA, et al., (2012). Seasonal dynamics of Synechococcus and Thaumarchaeal microbial populations in Monterey Bay resolved in real time with remote in situ instrumentation. *The ISME Journal*, 6: 513-523.

Suzuki MT, Taylor LT, DeLong EF. (2000). Quantitative analysis of small-subunit rRNA genes in mixed microbial populations via 5'-nuclease assays. *Appl Environ Microbiol* 66: 4605–4614.

Ussler W III, Preston C, Tavormina P, Pargett D, Jensen S, Roman B, *et al.* (2013). Autonomous Application of Quantitative PCR in the Deep Sea: In Situ Surveys of Aerobic Methanotrophs Using the Deep-Sea Environmental Sample Processor. *Environ Sci Technol* **47**:9339–9346.

Varaljay VA, Robidart J, Preston CM,  Gifford SM, Durham BP, Burns AS, et al.,(2015). Single-Taxon Field Measurements of Bacterial Gene Regulation Controlling DMSP Fate. *The ISME Journal*, 9: 1677–1686.

Wankel SD, Germanovich LN, Lilley MD, Genc G, DiPerna CJ, Bradley AS, *et al.* (2011). Influence of subsurface biosphere on geochemical fluxes from diffuse hydrothermal fluids. *Nature Geosci* **4**:1–8.

Yamahara KM, Demir-Hilton E, Preston CM, Marin III R, Pargett D, Roman B, et al. (2015). Simultaneous monitoring of fecal indicators and harmful algae using an in in situ auntomous sensor. *Letters in Applied Microbiology*, 61130-138.
