# Supplementary figures and images for: Co-registered Geochemistry and Metatranscriptomics Reveal Unexpected Distributions of Microbial Activity within a Hydrothermal Vent Field
Source: Front Microbiol. 2017 Jun 13;8:1042. doi: 10.3389/fmicb.2017.01042 (PMC5468400; doi:10.3389/fmicb.2017.01042)

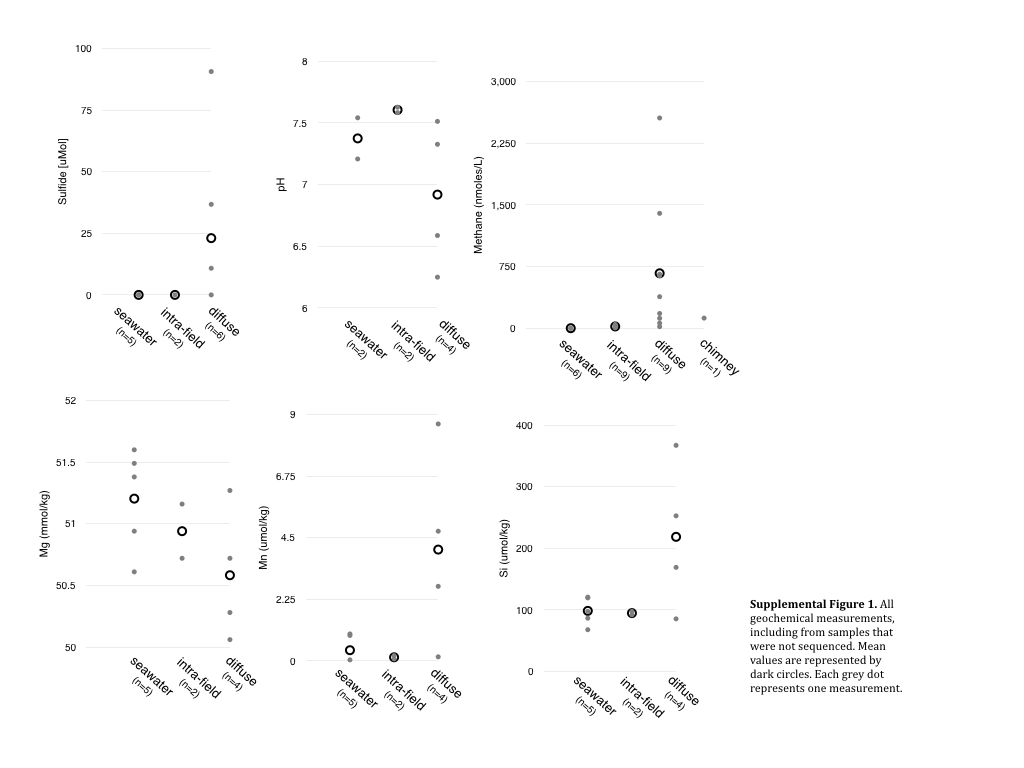

Supplement: Supplementary file 8 [file Image1.JPEG]

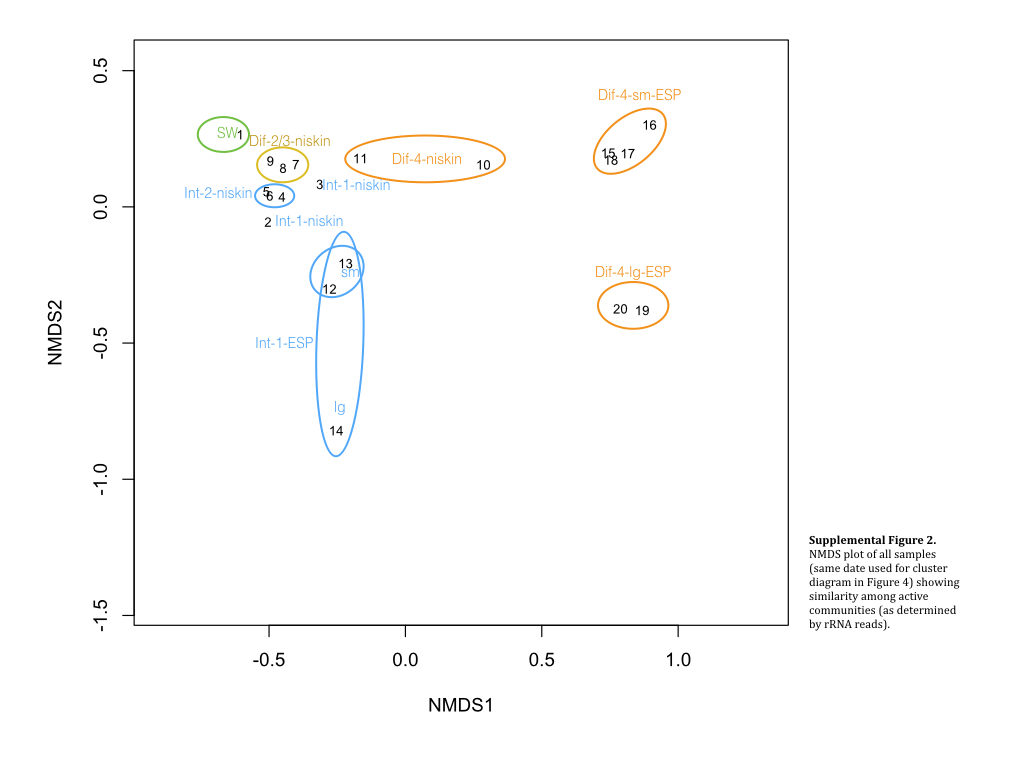

Supplement: Supplementary file 9 [file Image2.JPEG]

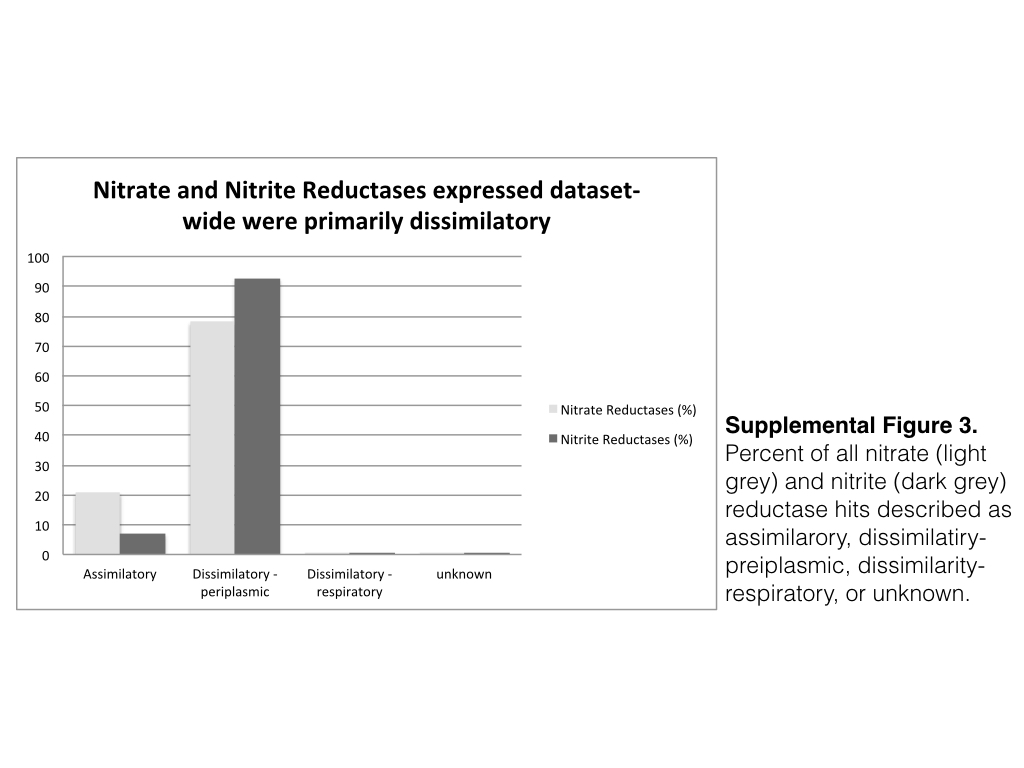

Supplement: Supplementary file 10 [file Image3.JPEG]

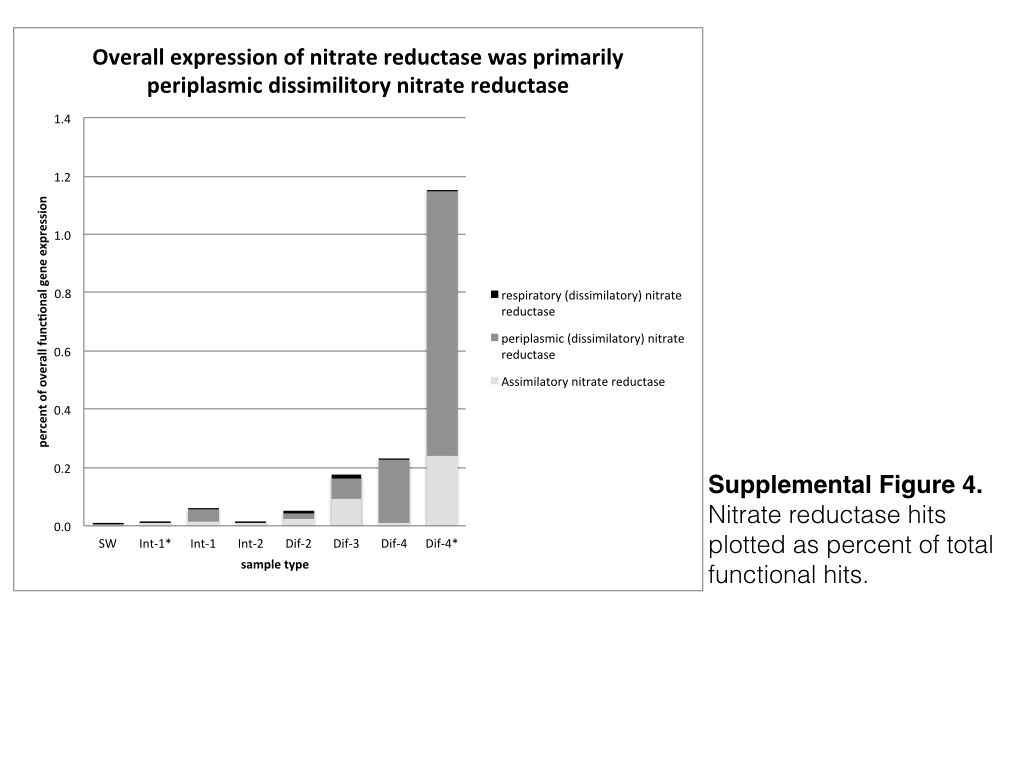

Supplement: Supplementary file 11 [file Image4.JPEG]

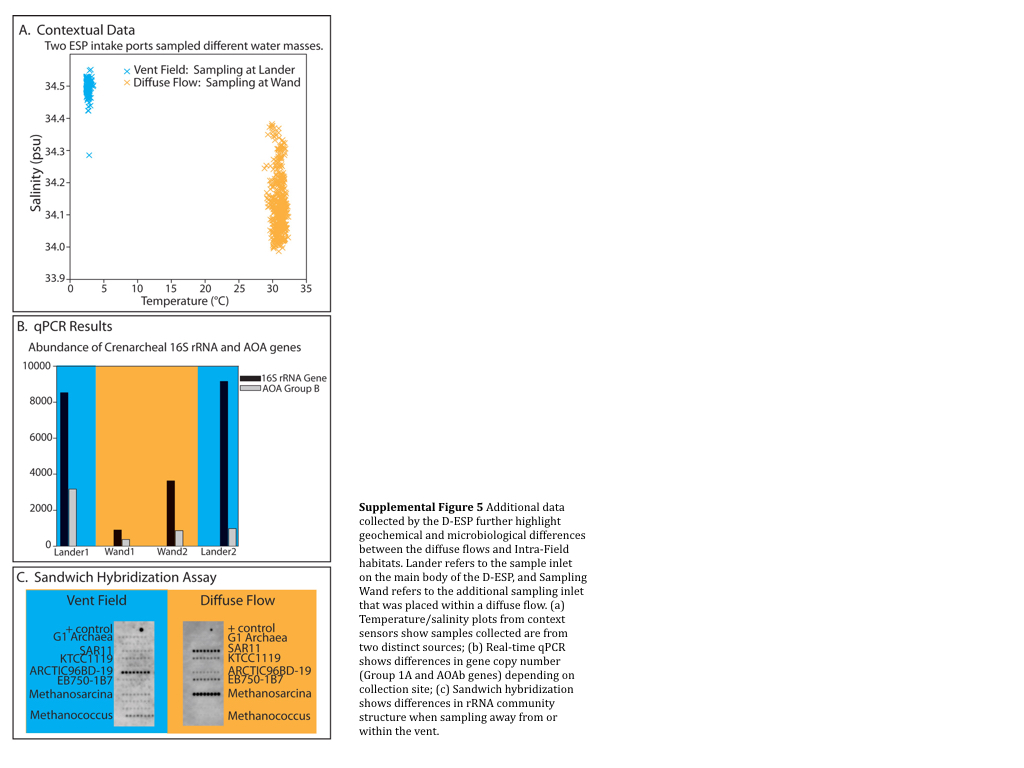

Supplement: Supplementary file 12 [file Image5.jpeg]
